# Dispersive surface-response formalism to address nonlocality in extreme plasmonic field confinement

**DOI:** 10.1515/nanoph-2023-0178

**Published:** 2023-06-21

**Authors:** Antton Babaze, Tomáš Neuman, Ruben Esteban, Javier Aizpurua, Andrei G. Borisov

**Affiliations:** Materials Physics Center CSIC-UPV/EHU, Paseo Manuel de Lardizabal 5, 20018, Donostia-San Sebastián, Spain; Donostia International Physics Center DIPC, Paseo Manuel de Lardizabal 4, 20018, Donostia-San Sebastián, Spain; Department of Electricity and Electronics, FCT-ZTF, UPV-EHU, 48080 Bilbao, Spain; Institut des Sciences Moléculaires d’Orsay, UMR 8214 CNRS-Université Paris-Saclay, Bât. 520, 91405 Orsay Cedex, France

**Keywords:** Feibelman parameters, nonlocality, plasmonics, quantum surface effects, surface response, time-dependent density functional theory

## Abstract

The surface-response formalism (SRF), where quantum surface-response corrections are incorporated into the classical electromagnetic theory *via* the Feibelman parameters, serves to address quantum effects in the optical response of metallic nanostructures. So far, the Feibelman parameters have been typically obtained from many-body calculations performed in the long-wavelength approximation, which neglects the nonlocality of the optical response in the direction parallel to the metal–dielectric interface, thus preventing to address the optical response of systems with extreme field confinement. To improve this approach, we introduce a *dispersive* SRF based on a general Feibelman parameter *d*_⊥_(*ω*, *k*_‖_), which is a function of both the excitation frequency, *ω*, and the wavenumber parallel to the planar metal surface, *k*_‖_. An explicit comparison with time-dependent density functional theory (TDDFT) results shows that the *dispersive* SRF correctly describes the plasmonic response of planar and nonplanar systems featuring extreme field confinement. This work thus significantly extends the applicability range of the SRF, contributing to the development of computationally efficient semiclassical descriptions of light–matter interaction that capture quantum effects.

## 1 Introduction

The excitation of plasmon resonances in metallic nanostructures has attracted great interest owing to the capability of nanoscale plasmonic systems to enhance and squeeze incident electromagnetic fields at subwavelength regions. Plasmon resonances have been widely used in a variety of spectroscopy and microscopy techniques such as surface-enhanced Raman spectroscopy [1], surface-enhanced fluorescence [2], [3], [4], or single-molecule imaging [5, 6], and enable promising applications in biomedicine [7], [8], [9], energy storage [10], [11], [12], and nonlinear optics [13, 14], among others. Miniaturization of plasmonic devices pushes light–matter interaction to the limit, reaching situations where quantum many-body phenomena can influence the optical properties of a system [15– 22]. In these extreme situations, classical descriptions based on local dielectric functions of materials characterized by abrupt interfaces are no longer valid [23, 24]. Thus, theoretical approaches incorporating nonlocality [25– 30], realistic electron density distribution at the interfaces [31– 34], and electron tunneling [35, 36] are required to describe the optical properties of plasmonic structures with very small characteristic dimensions. In this context, time-dependent density functional theory (TDDFT) is often used because it accounts for the quantum nature of the electron dynamics from first principles [37– 41]. However, because of its computational cost [42, 43], TDDFT is limited to addressing systems with a relatively low number of electrons in the relevant dimension(s) of the nanostructure (typically a few-nanometers structures). As a consequence, computationally less-demanding semiclassical approaches have been developed to capture quantum effects within the framework of classical electrodynamics [33, 44– 55].

Among the semiclassical approaches, the surface-response formalism (SRF) [23, 56], [57], [58], [59] has prompted great interest in the nanophotonics community over the last few years. The SRF is based on the theoretical framework proposed by Peter Feibelman in the eighties [60] to describe the interaction of electromagnetic radiation with planar metal surfaces. This formalism introduces quantum surface-response corrections into the classical Maxwell’s theory [61] through the so-called Feibelman parameters. As a consequence, quantum effects such as surface-enabled Landau damping [62], [63], [64], spill-out of the induced electron density [34, 51, 54], and nonlocal dynamical screening [27, 28, 57, 65] are accounted for. The Feibelman parameters, commonly denoted as *d*_⊥_ and *d*_‖_, are complex-valued functions characterizing the dynamically induced charges (*d*_⊥_) and the parallel-to-the-surface component of the induced currents (*d*_‖_) at the metal–dielectric interface [32].

In the literature, the practical implementation of the SRF within the Feibelman theory typically involves two approximations. First, *d*_‖_ is assumed to be zero, which is a reasonable approach given that *d*_‖_ strictly vanishes for charge-neutral planar interfaces [32, 56, 66]. Second, the nonlocality of the optical response in the direction parallel to the metal–dielectric interface is neglected. Within this long-wavelength approximation, it is considered that the characteristic wavenumber *k*_‖_ parallel to the metal surface is negligible, so that the Feibelman parameter *d*_⊥_(*ω*) is exclusively a function of the excitation frequency, *ω*. Considering the long-wavelength limit with *k*_‖_ = 0 reduces the computational effort in obtaining *d*_⊥_(*ω*) from quantum calculations, and simplifies the implementation of the SRF in existing numerical tools that solve Maxwell’s equations in nanophotonics, as employed in a number of recent studies [59, 67–74]. In what follows, we refer to *d*_⊥_(*ω*) as the *nondispersive* Feibelman parameter.

Using the nondispersive Feibelman parameter is reasonable when the nonlocality of the optical response in the direction perpendicular to the metal surface dominates, i.e., when the characteristic length scale of the optical field variation along the surface is relatively large, 2*π*/*k*_‖_ ≫ *λ*_*F*_, where *λ*_*F*_ is the Fermi wavelength of the metal. This is the case of e.g. typical individual nanoparticles subjected to plane-wave illumination. However, for localized probes in proximity of metal nanoparticle surfaces, where high-order plasmonic modes can be excited, the long-wavelength approximation to *d*_⊥_(*ω*) is compromised [75]. Indeed, in such situations, multipolar plasmon modes characterized by localized surface charges that rapidly vary along the nanoparticle surface are involved. This is analogous to exciting surface plasmons with large transverse wavenumber *k*_‖_ at planar metal–dielectric interfaces, and therefore requires going beyond the long-wavelength limit of *d*_⊥_.

**Figure 1:**
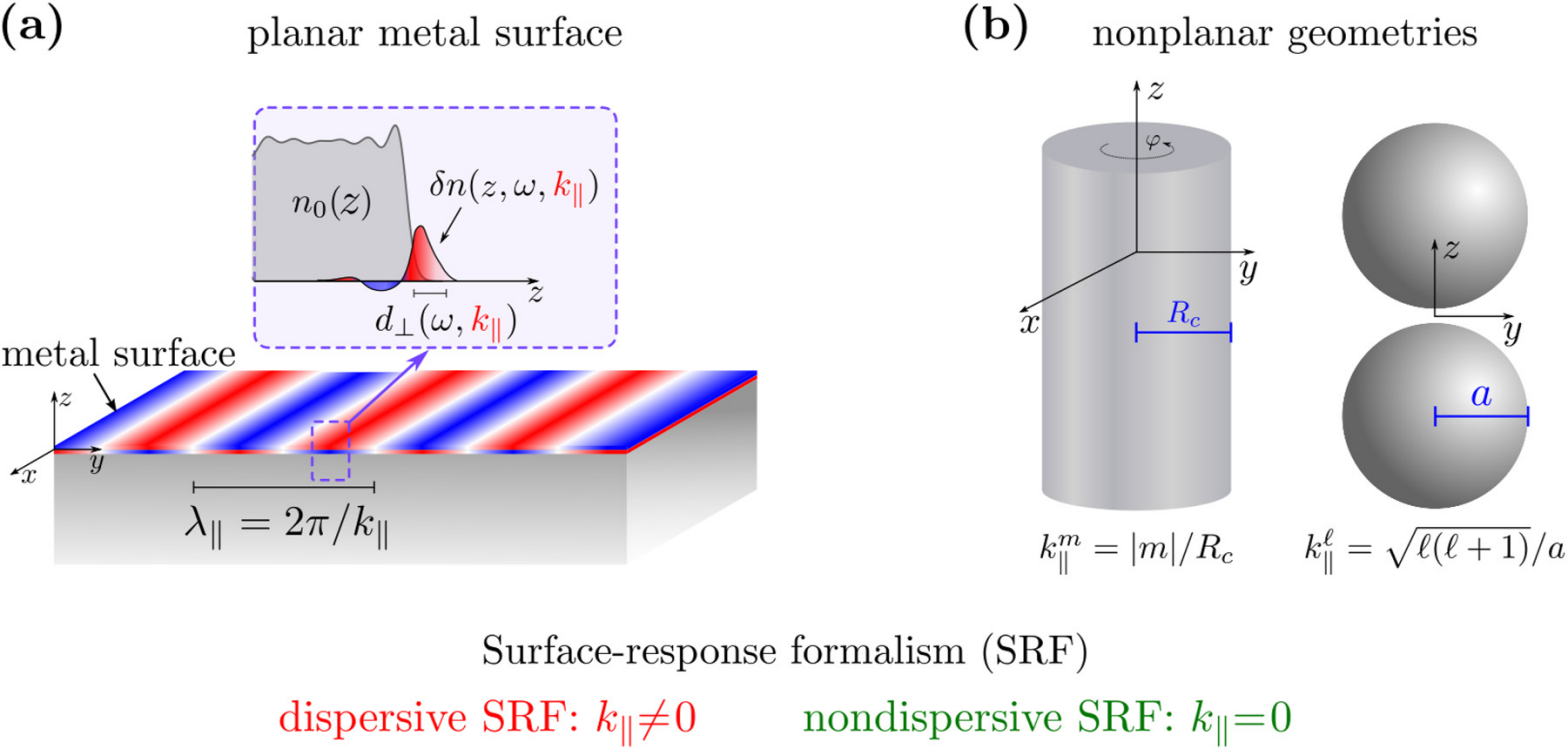
Dispersive surface-response formalism for plasmonic systems. (a) Sketch of the dispersive surface-response formalism (SRF) employed in this work. The real part of the dispersive Feibelman parameter *d*_⊥_(*ω*, *k*_‖_) accounts for the position of the centroid of the induced electron density *δn*(*z*, *ω*, *k*_‖_) relative to the geometrical metal surface, and the imaginary part is related to surface-enabled Landau damping. The dispersive Feibelman parameter *d*_⊥_(*ω*, *k*_‖_) is a function of the excitation frequency, *ω*, and the wavenumber parallel to the planar metal surface, *k*_‖_ = 2*π*/*λ*_‖_ (with *λ*_‖_ the wavelength of the nonretarded surface plasmon). The nondispersive SRF assumes that *k*_‖_ = 0 (*λ*_‖_ → ∞), so that *d*_⊥_ is a function of *ω* exclusively. *n*_0_(*z*) represents the equilibrium electron density. (b) Sketch of nonplanar structures considered in this work. Left: cylindrical metallic nanowire with radius *R*_*c*_, infinite along the *z*-axis, with *φ* the azimuth angle. The system possesses rotational and translational symmetry with respect to the *z*-axis. The equivalence between *k*_‖_ and |*m*|/*R*_*c*_ is used to implement the dispersive SRF in the cylindrical nanowire, $k_{\|}^{m}=|m|/R_{c}$ (with *m* the magnetic quantum number of a particular plasmonic mode). Right: a dimer of two identical spherical metallic nanoparticles of radius *a*. An effective wavenumber $k_{\|}^{\ell }=\sqrt{\ell \left(\ell +1\right)}/a$ is assigned to implement the dispersive SRF in the spherical nanoparticles (with *ℓ* the angular momentum quantum number of the plasmonic mode). All the structures are considered to be made of sodium, characterized using a Wigner–Seitz radius *r*_*s*_ = 4 *a*_0_ (with *a*_0_ = 0.0529 nm the Bohr radius).

In this work, we demonstrate that considering the nonlocality of the optical response in the direction parallel to the metal surface significantly improves the performance of the SRF. While earlier theory addresses the role of nonlocality by introducing an *ad hoc* dipole layer [58], we base our approach on the implementation of a *dispersive* Feibelman parameter *d*_⊥_(*ω*, *k*_‖_) that is a function of *ω* and *k*_‖_, providing a direct connection of the theory to the microscopic quantum aspects of screening. The numerical values of this parameter are obtained from linear-response frequency-domain TDDFT calculations using a planar free-electron metal slab. We then show that the dispersive SRF based on the same set of *d*_⊥_(*ω*, *k*_‖_) can be used to describe the plasmon modes and optical response not only for planar surfaces, but also for nonplanar geometries. In particular, the method proposed in this work is relevant for situations where plasmon resonances with large transverse momenta (small plasmon wavelength) are excited. We test the validity of the dispersive SRF by using quantum many-body TDDFT simulations as a reference for a planar metal surface (Figure 1a) as well as for canonical plasmonic nanostructures such as cylindrical metallic nanowires, small spherical metal nanoparticles, and plasmonic gaps as exemplified by a spherical dimer (Figure 1b). In all the systems studied, the dispersive SRF reproduces the TDDFT results, demonstrating its validity to capture the effects linked to nonlocality of dynamical screening along the metal surface. The theoretical framework proposed here provides significant conceptual advances toward the implementation of efficient semiclassical approaches that adequately account for quantum effects in the optical response of plasmonic systems.

## 2 Methodology

To obtain the dispersive Feibelman parameter, *d*_⊥_(*ω*, *k*_‖_), we employ linear-response frequency-domain TDDFT calculations for a planar metal slab. The adiabatic local-density approximation (ALDA) with the exchange–correlation potential given by Gunnarsson and Lunqvist [76] is used. The metal slab is infinite along the (*x*, *y*)-plane and has a finite thickness *L* in the *z*-direction. The metal surface is located in the (*x*, *y*, *z* = 0) plane, with vacuum at *z* > 0 (see Figure 1a). The electronic structure of the slab is described within the jellium model of free-electron metals [77–81] using a Wigner–Seitz radius *r*_*s*_ = 4 *a*_0_ that corresponds to sodium (*a*_0_ = 0.0529 nm is the Bohr radius). The bulk plasma frequency is *ω*_*p*_ = 5.89 eV, and the nonretarded surface plasmon frequency for *k*_‖_ = 0 is $\omega_{\text{SP}}=\omega_{p}/\sqrt{2}=4.16$ eV. The choice of this material allows for a direct comparison between our results and those obtained in recent works using the nondispersive SRF [67, 70, 75]. Further details can be found in ref. [32] and in Section S1 of Supplementary Material. The methodology employed in this work is well suited for simple free-electron metals. For noble metals, interband transitions involving bound *d*-band electrons contribute to the screening of the electromagnetic field and thus affect the optical response. The present approach could be extended to these situations by mimicking the effect of *d*-electrons with the inclusion of a polarizable background [65, 71, 82].

Since the nonlocality of the metal response in the direction parallel to the surface is important in geometries confining fields at typical length scales comparable to that of the Fermi wavelength of electrons [58, 75] (i.e., within the nm range), we use the nonretarded approximation within the linear-response theory. The optical response of the metal slab to a time-dependent external potential $V_{\text{ext}}\left(z,r_{\|},t\right)=V_{\text{ext}}\left(z,\omega ,k_{\|}\right){\text{e}}^{\text{i}k_{\|}r_{\|}-i\omega t}$ oscillating at frequency *ω* is determined from the induced electron density $\delta n\left(z,r_{\|},t\right)=\delta n\left(z,\omega ,k_{\|}\right){\text{e}}^{\text{i}k_{\|}r_{\|}-i\omega t}$ , where **r**_‖_ is the component of the position vector parallel to the metal surface, and **k**_‖_ is the parallel wavevector (*k*_‖_ = |**k**_‖_|).

Within the many-body formalism, *δn*(*z*, *ω*, *k*_‖_) is given by

(1) $$\delta n\left(z,\omega ,k_{\|}\right)=-\int dz^{\prime }\chi \left(z,z^{\prime },\omega ,k_{\|}\right)V_{\text{ext}}\left(z^{\prime },\omega ,k_{\|}\right),$$

where $\chi \left(z,z^{\prime },\omega ,k_{\|}\right)$ is the many-body response function (see further details in Section S1 of Supplementary Material). Here we use the Fourier component of the external potential given by

(2) $$V_{\text{ext}}\left(z,\omega ,k_{\|}\right)=\frac{2\pi }{k_{\|}}e^{k_{\|}z},$$

which exponentially decays within the metal (*z* < 0). Note that, up to a multiplying factor, Eq. (2) represents the asymptotic behavior of the potential created by any charge distribution located in vacuum far from the surface, since the plane-wave decomposition of such potential can be obtained from the Green’s function *G*(**r** − **r**′) corresponding to the potential of a point charge located at **r**′:

(3) $$G\left(r-r^{\prime }\right)=\frac{1}{{\left(2\pi \right)}^{2}}\iint {\text{d}}^{2}k_{\|}\;\frac{2\pi }{k_{\|}}{\text{e}}^{\text{i}k_{\|}\left(r_{\|}-r_{\|}^{\prime }\right)-k_{\|}\left|z-z^{\prime }\right|}.$$

In this respect, within the linear-response regime the induced electron density *δn*(*z*, *ω*, *k*_‖_) given by Eq. (1) is independent of the specific form of the external potential that excites the system.

The dispersive Feibelman parameter *d*_⊥_(*ω*, *k*_‖_) obtained from

(4) $$d_{⊥}\left(\omega ,k_{\|}\right)=\frac{\int \;dz\;z\;\delta n\left(z,\omega ,k_{\|}\right)}{\int \;dz\;\delta n\left(z,\omega ,k_{\|}\right)}$$

is therefore a characteristic of the surface response inherent to the specific metal and the surrounding material [32]. In contrast to the nondispersive parameter [59, 67, 70], [71], [72], [73], the dispersive *d*_⊥_(*ω*, *k*_‖_) introduced in Eq. (4) depends on the wavenumber *k*_‖_ parallel to the surface, and thus explicitly accounts for the nonlocality of the many-body response and screening in this direction. Other definitions different from Eq. (4) that express the Feibelman parameter in terms of the electromagnetic fields and nonlocal dielectric functions have also been used. A discussion on the equivalence between Eq. (4) and other definitions can be found in refs. [32, 60, 83].

As follows from Eq. (2), the *z*-extension of the external potential within the metal slab increases with decreasing *k*_‖_. In our calculations (where *k*_‖_ ≠ 0), we use a slab with thickness *L* = 870 *a*_0_ (≈46nm) , large enough to avoid the interaction between the charges induced at the opposite surfaces of the slab. For *k*_‖_ = 0 the slab geometry is not applicable, and we rely for this case on the nondispersive parameter *d*_⊥_(*ω*) calculated by Christensen et al. [67] for the semi-infinite metal characterized by the same *r*_*s*_ as that used here.

## 3 Results and discussion

### 3.1 Dispersive Feibelman parameter

**Figure 2:**
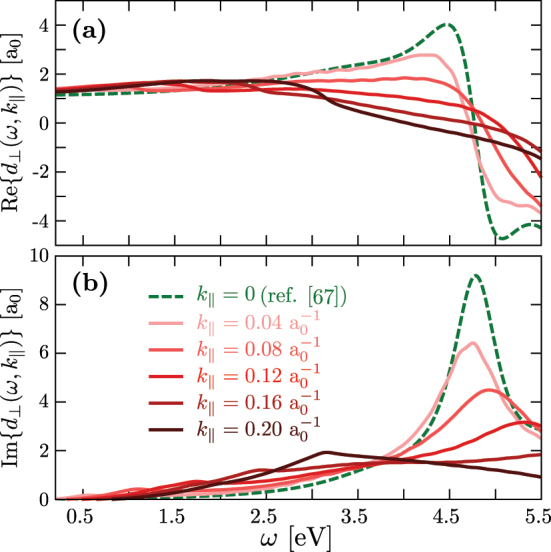
Dispersive Feibelman parameter. (a) Real part of the dispersive Feibelman parameter, $\text{Re}\left\{d_{⊥}\left(\omega ,k_{\|}\right)\right\}$ , calculated here within the linear-response frequency-domain TDDFT as a function of frequency *ω*, for selected values of *k*_‖_ as indicated in panel b. We also show the parameter $\text{Re}\left\{d_{⊥}\left(\omega \right)\right\}$ obtained in ref. [67] by Christensen et al. under the long-wavelength approximation (dashed green line). (b) Same as in (a) but for the imaginary part of the dispersive Feibelman parameter, $\text{Im}\left\{d_{⊥}\left(\omega ,k_{\|}\right)\right\}$ .

Figure 2 shows the real part $\text{Re}\left\{d_{⊥}\left(\omega ,k_{\|}\right)\right\}$ (panel a) and the imaginary part $\text{Im}\left\{d_{⊥}\left(\omega ,k_{\|}\right)\right\}$ (panel b) of the dispersive Feibelman parameter as a function of the frequency *ω* and the parallel-to-the-surface wavenumber *k*_‖_. With the definition given by Eq. (4), the real part of the Feibelman parameter determines the position of the centroid of the induced electron density with respect to the geometrical metal surface (jellium edge) located at *z* = 0 (see inset in Figure 1a). The imaginary part of the Feibelman parameter is related to the surface loss function and thus to the energy absorption by electronic excitations at the surface [32, 84]. These electronic excitations mainly consist of the Bennett plasmon [42, 85], characterized by the presence of induced electron density of opposite sign across a single metal–vacuum interface (see Figure 2 in ref. [51]), and electron–hole pair excitations involved in the Landau damping mechanism of plasmons [62, 86, 87]. A detailed discussion of the frequency dependence of the Feibelman parameter within the long-wavelength limit *k*_‖_ = 0 can be found in ref. [32]. Therefore, we only discuss briefly the main features of *d*_⊥_(*ω*, *k*_‖_) with particular emphasis on its *k*_‖_ dependence.

For small *k*_‖_, the TDDFT results in Figure 2 are consistent with earlier findings for *k*_‖_ = 0 [67], with *d*_⊥_(*ω*, *k*_‖_) featuring a complex Lorentzian-like resonance at *ω* ∼ 4.6 eV. This resonance is associated with the excitation of the Bennett plasmon at *ω* ∼ 0.8*ω*_*p*_ [42, 85, 88]. With increasing *k*_‖_, the Bennett plasmon resonance broadens, blueshifts, and gradually disappears. $\text{Im}\left\{d_{⊥}\left(\omega ,k_{\|}\right)\right\}$ is then dominated for large *k*_‖_ by electron–hole pair excitations that extend over the whole frequency range addressed here. At low frequencies, *ω* ≲ 1 eV, the optical response of the system is close to that of an ideal metal, and becomes nearly independent of *k*_‖_ and *ω*. In this situation, $\underset{\omega \to 0}{\lim}\left[\text{Re}\left\{d_{⊥}\left(\omega ,k_{\|}\right)\right\}\right]\approx 1.2\;a_{0}$ determines the position of the electrostatic image plane of the free-electron metal with respect to its jellium edge.

Importantly, in the frequency range *ω* ∼ 2 − 4.5 eV relevant for plasmon excitations in different structures (see below), the following trends can be observed with increasing *k*_‖_:

–
$\text{Re}\left\{d_{⊥}\left(\omega ,k_{\|}\right)\right\}$ decreases and even changes the sign from positive to negative. This indicates that the centroid of the induced electron density shifts inwards the metal for large *k*_‖_.
–
$\text{Im}\left\{d_{⊥}\left(\omega ,k_{\|}\right)\right\}$ overall increases in this range. This tendency reflects a more efficient decay of the plasmon into electron–hole pair excitations *via* surface-enabled Landau damping [42, 62, 84, 86, 89], [90], [91].

In general, the results in Figure 2 demonstrate a strong dependence of the dispersive Feibelman parameter *d*_⊥_(*ω*, *k*_‖_) on *k*_‖_ due to nonlocal dynamical screening. Thus, one can expect that the dispersive and nondispersive SRF yield very different results in situations that involve a rapid variation of plasmon-induced charges along the metal–vacuum interface. In the following sections, we analyze the limitations of the nondispersive SRF and illustrate the good performance of the dispersive SRF using the example of canonical plasmonic nanostructures described semiclassically within the SRF and quantum mechanically within TDDFT. Our results show that the dispersive SRF correctly captures quantum surface effects within a computationally efficient semiclassical framework.

### 3.2 Validation of the dispersive SRF

#### 3.2.1 Nonlocal dynamical screening of a planar metal surface

As a first application of the SRF, we consider the plasmon propagating at the interface between a planar metal surface and vacuum. The surface plasmon frequency dispersion *ω*_*s*_(*k*_‖_) with parallel wavenumber can be obtained within TDDFT from the surface loss function Im $\left\{g\left(\omega ,k_{\|}\right)\left.\right)\right\}$ , which reveals the rate at which the electron–hole pair excitations at a given frequency *ω* are produced at the surface (including both single-particle and collective excitations) [32, 84]. Here, the so-called surface response function *g*(*ω*, *k*_‖_) is given by

(5) $$g\left(\omega ,k_{\|}\right)=\int dz\;\delta n\left(z,\omega ,k_{\|}\right)e^{k_{\|}z},$$

where the induced electron density *δn*(*z*, *ω*, *k*_‖_) is obtained within TDDFT considering the slab geometry introduced in the previous section.

**Figure 3:**
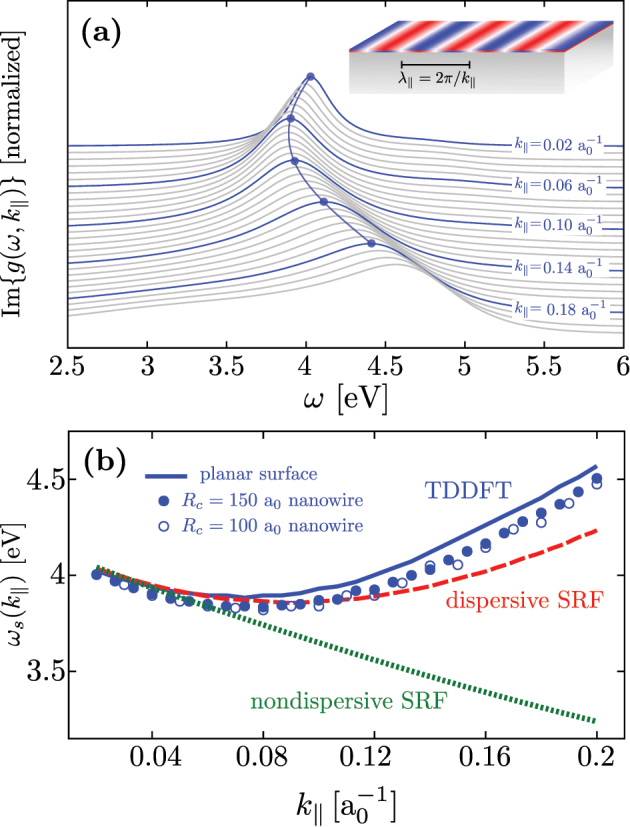
Surface plasmon dispersion. (a) Surface loss function, Im $\left\{g\left(\omega ,k_{\|}\right)\right\}$ , of a *L* = 870 *a*_0_ thick jellium metal slab calculated within the linear-response frequency-domain TDDFT as a function of the excitation frequency, *ω*. The results obtained for different values of the wavenumber *k*_‖_ parallel to the surface, ranging from $k_{\|}=0.02\;a_{0}^{-1}$ (top) to $k_{\|}=0.2\;a_{0}^{-1}$ (bottom), are vertically offset for clarity. The blue curves are labeled with the corresponding value of *k*_‖_, and the gray curves correspond to intermediate values. (b) Dispersion relationship of the frequency of the surface plasmon, *ω*_*s*_, as a function of *k*_‖_. Blue solid line: linear-response frequency-domain TDDFT results (defined as the frequency at which Im $\left\{g\left(\omega ,k_{\|}\right)\right\}$ is maximum). Red dashed line: dispersive SRF results. Green dotted line: nondispersive SRF results. For the dispersive and nondispersive SRF results Eq. (6) is used. The frequency *ω*_*m*_ of the localized multipolar plasmon of order *m* sustained by a cylindrical metallic nanowire of radius *R*_*c*_ = 150 *a*_0_ (filled circles) and *R*_*c*_ = 100 *a*_0_ (hollow circles) are shown as a function of the effective wavenumber $k_{\|}^{m}=|m|/R_{c}$ .

Figure 3a shows the frequency dependence of the surface loss function, Im $\left\{g\left(\omega ,k_{\|}\right)\right\}$ , calculated for a *L* = 870 *a*_0_ thick jellium metal slab. Results are obtained for different values of the wavenumber *k*_‖_ parallel to the surface, as indicated in the figure. Our findings nicely reproduce the general behavior of propagating plasmon resonances documented in detail for simple metal surfaces (here sodium surface) [32, 84, 85, 92], [93], [94], [95]. In particular, with increasing *k*_‖_, the frequency of the surface plasmon resonance (defined from the maximum of Im $\left\{g\left(\omega ,k_{\|}\right)\right\}$ ) first redshifts at low *k*_‖_ and, after reaching a minimum value, it continuously blueshifts for higher *k*_‖_. The width of the plasmon resonance increases with increasing *k*_‖_ because of the enhancement of surface-enabled Landau damping [32, 84, 86, 91, 93].

The frequency dispersion *ω*_*s*_(*k*_‖_) featured by the plasmon resonances in Figure 3a is intimately related to the *k*_‖_-dependence of the position of the centroid of the induced electron density (given by Re{*d*_⊥_(*ω*, *k*_‖_)}). Indeed, for low *k*_‖_ the induced electron density is shifted outward from the geometrical metal surface (Re{*d*_⊥_(*ω*, *k*_‖_)} > 0 in Figure 2a), i.e., toward the region of lower electron density, which produces the redshift of *ω*_*s*_(*k*_‖_) with respect to $\omega_{\text{SP}}=\omega_{p}/\sqrt{2}=4.16$ eV (see Eq. (6) below) [32]. In contrast, for high *k*_‖_, the plasmon-induced electron density is shifted inward the geometrical surface (Re{*d*_⊥_(*ω*, *k*_‖_)} < 0), thus producing a blueshift of *ω*_*s*_(*k*_‖_) relative to *ω*_SP_.

We continue the quantitative analysis of the plasmon dispersion in Figure 3b, where we show the resonant plasmon frequency *ω*_*s*_(*k*_‖_) obtained from TDDFT calculations (blue solid line) and compare it with dispersive (red dashed line) and nondispersive (green dotted line) SRF results. To find the *ω*_*s*_(*k*_‖_) dispersion using the SRF based on the Feibelman parameters, we describe the dielectric function of the metal with a Drude model, and self-consistently solve the following transcendental equation [32, 60, 65, 84, 96], [97], [98],

(6) $$\omega_{s}\left(k_{\|}\right)=\omega_{\text{SP}}\left(1-\frac{k_{\|}}{2}\text{Re}\left\{d_{⊥}\left(\omega_{s},k_{\|}\right)\right\}\right),$$

where $\omega_{\text{SP}}=\omega_{p}/\sqrt{2}=4.16$ eV, and |*k*_‖_Re{*d*_⊥_(*ω*_*s*_, *k*_‖_)}|≪ 1 is assumed (see details in Section S6 of Supplementary Material). Throughout this work, we use Re{*d*_⊥_(*ω*, *k*_‖_)} as obtained from the TDDFT results reported in Figure 2a for the dispersive SRF, while for the nondispersive model we use *d*_⊥_(*ω*) as calculated by Christensen et al. [67].

Figure 3b shows that the dispersive SRF and TDDFT results are in good agreement within the broad range of *k*_‖_ values considered here. The dispersive SRF reproduces the redshift followed by blueshift of *ω*_*s*_(*k*_‖_) with increasing *k*_‖_ as predicted by TDDFT. For high *k*_‖_ values, the quantitative agreement between the two sets of results worsens. This may be attributed to the short lifetime of plasmon modes at high *k*_‖_. The resonant structures in the surface loss function become broad and asymmetric so that the resonant frequencies are ill defined. Additionally, higher-order terms of the induced electron density near the surface beyond the leading dipolar contribution accounted for with *d*_⊥_(*ω*, *k*_‖_) may gain in importance in such situations. In sheer contrast, the nondispersive SRF fails to describe the entire *ω*_*s*_(*k*_‖_) dependence, since it predicts a continuous redshift of *ω*_*s*_(*k*_‖_) with increasing *k*_‖_. Therefore, the nondispersive SRF is only accurate for small values of $k_{\|}≲0.06\;a_{0}^{-1}$ , where the long-wavelength approximation is well justified. Figure 3b thus demonstrates that using the dispersive Feibelman parameter *d*_⊥_(*ω*, *k*_‖_) within the SRF allows us to correctly capture the main nonlocal effect associated with the dependence of the dynamical screening on *k*_‖_.

#### 3.2.2 Localized multipolar plasmon resonances in a cylindrical nanowire

We study next the applicability of the dispersive SRF to address nonlocal effects in the optical response and plasmon resonances of nonplanar plasmonic nanostructures used in a variety of applications in nanophotonics. To this end, we first consider localized multipolar plasmons (LMP) sustained by cylindrical metallic nanowires of radii *R*_*c*_ = 75 − 150 *a*_0_ (≈4 − 8 nm), infinite along the *z*-axis (see Figure 1b). The nanowire is described within the same jellium model as that used for the metal slab. The reference quantum calculations are performed using real-time ALDA-TDDFT as implemented in prior works [27, 28, 99], [100], [101], [102] (see Section S2 of Supplementary Material for further details). The excitation of LMPs evolving in the (*x*, *y*)-plane determines the optical response of the nanowire for an incident electromagnetic wave polarized such that its electric field is perpendicular to the nanowire *z*-axis. The system is translationally invariant along the *z*-axis, and under the illumination conditions considered here, the wavevector describing the plasmon propagation along the nanowire is *k*_*z*_ = 0 (see Supplementary Material). Consistent with the symmetry, the LMPs can be characterized by their multipole order *m* related to the e^i*mφ*^ dependence on the azimuth angle *φ* of the potentials, electric near fields, and induced charges.

**Figure 4:**
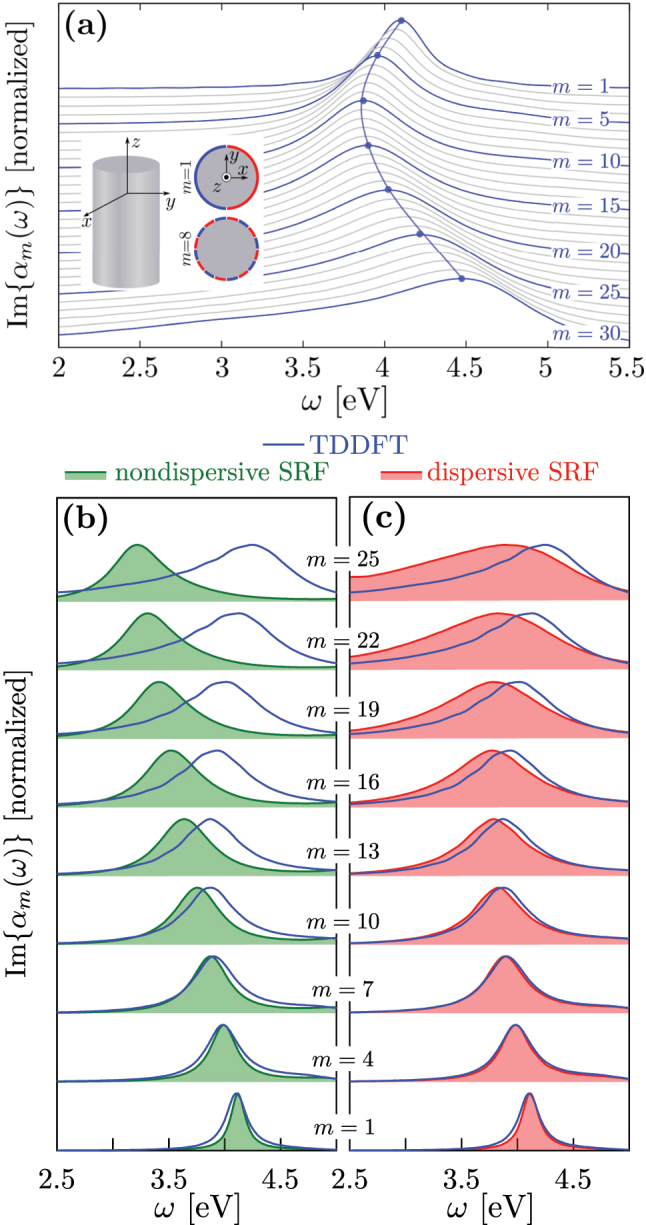
Imaginary part of the multipololar polarizability of order *m*, Im{*α*_*m*_(*ω*)}, of a Na nanowire of radius *R*_*c*_ = 150 *a*_0_ (≈8 nm). Results are shown as a function of the frequency of the excitation, *ω*, for different values of the magnetic quantum number *m*. Each spectrum of Im{*α*_*m*_(*ω*)} is normalized to 1 at its maximum, and vertically offset for clarity. (a) Real-time TDDFT results with *m* ranging from *m* = 1 (top) to *m* = 30 (bottom). The blue curves are labeled with the corresponding value of *m*, and the gray curves correspond to intermediate values. (b, c) Nondispersive SRF (b, green filled curves), dispersive SRF (c, red filled curves) and real-time TDDFT (blue lines) results with *m* ranging from *m* = 1 (bottom) to *m* = 25 (top). The dispersive and nondispersive SRF results are obtained from Eq. (7).

To analyze the spectral properties of the LMPs of order *m*, we show in Figure 4a the TDDFT results of the imaginary part of the multipolar polarizability *α*_*m*_(*ω*) per unit length along the *z*-axis of a cylindrical nanowire of radius *R*_*c*_ = 150 *a*_0_ (see Section S4 in Supplementary Material). Results are presented as a function of frequency for different values of *m* ranging from *m* = 1 (top) to *m* = 30 (bottom). The resonances in Im{*α*_*m*_(*ω*)} are associated with the excitation of the corresponding LMP of order *m*. Interestingly, their general behavior with *m* is similar to that of the propagating surface plasmon with *k*_‖_ (see Figure 3a). Indeed, the resonant frequency *ω*_*m*_ first redshifts starting with *m* = 1, and, after reaching a minimum value at *m* ≈ 10, it continuously blueshifts. Moreover, similarly to the situation of the planar surface, the width of the LMP resonances increases with increasing *m* because of the enhancement of Landau damping.

The close correspondence between the *m*-dependence of the frequency of the LMPs of the nanowire and the *k*_‖_-dispersion of the surface plasmon resonances at the planar metal–vacuum interface can be understood as follows. By introducing the coordinate *r*_‖_ along the circumference of the cylinder, *r*_‖_ = *R*_*c*_ *φ*, the angular dependence of the induced electric fields and surface electron densities of the LMPs transforms to $\exp\left(\text{i}m\varphi \right)\to \exp\left(\text{i}\frac{m}{R_{c}}\;r_{\|}\right)$ . Thus, the LMPs of the nanowire can be considered as surface plasmons confined around the cylinder circumference and characterized by a quantized wavenumber $k_{\|}^{m}=\frac{\left|m\right|}{R_{c}}$ [103] (see inset in Figure 4a). Also note that, when solving Laplace equation for the induced potential, $m^{2}/R_{c}^{2}$ at the nanowire and $k_{\|}^{2}$ in the slab geometry both are related to the variation of the quantities in the direction tangential to the metal surface.

To support the equivalence between *k*_‖_ and |*m*|/*R*_*c*_, we show in Figure 3b the plasmon frequency *ω*_*m*_ of cylinders with different radii as a function of $k_{\|}^{m}=|m|/R_{c}$ . The results obtained for *R*_*c*_ = 150 *a*_0_ (blue filled circles) and *R*_*c*_ = 100 *a*_0_ (blue hollow circles) fall on a universal curve very close to the plasmon dispersion relationship of the planar metal surface (blue solid line), thus confirming that an effective wavenumber $k_{\|}^{m}=|m|/R_{c}$ determines the optical response of the nanowire. Further results corroborating this finding are provided in Supplementary Material.

Owing to the equivalence $k_{\|}^{m}=|m|/R_{c}$ , the dispersive SRF can be straightforwardly applied to describe the nonlocal optical response of the cylindrical nanowire. The nonretarded multipolar polarizability *α*_*m*_(*ω*) of a cylindrical nanowire per unit length along the *z*-axis can be obtained within the SRF from (see Section S4 in Supplementary Material)

(7) $$\alpha_{m}\left(\omega \right)\propto \frac{\left(\varepsilon \left(\omega \right)-1\right)\left(1+\frac{\left|m\right|}{R_{c}}d_{⊥}\left(\omega ,k_{\|}=k_{\|}^{m}\right)\right)}{\varepsilon \left(\omega \right)+1-\left[\varepsilon \left(\omega \right)-1\right]\frac{\left|m\right|}{R_{c}}d_{⊥}\left(\omega ,k_{\|}=k_{\|}^{m}\right)},$$

where *ɛ*(*ω*) is the dielectric function of the metal described here with a Drude model (bulk plasma frequency *ω*_*p*_ = 5.89 eV and intrinsic damping parameter *γ*_*p*_ = 0.1 eV [75]). In Eq. (7), $d_{⊥}\left(\omega ,k_{\|}=k_{\|}^{m}\right)$ is the dispersive Feibelman parameter calculated in this work for a planar metal surface (Figure 2). For the sake of comparison, we also calculate *α*_*m*_(*ω*) using the nondispersive SRF where *d*_⊥_(*ω*, *k*_‖_) → *d*_⊥_(*ω*). Note that the LMP resonance frequencies within the SRF can be obtained from the poles of Eq. (7), which are exactly given by Eq. (6) using $k_{\|}=k_{\|}^{m}=|m|/R_{c}$ .

Figure 4b and c show the result of Im{*α*_*m*_(*ω*)} for a Na nanowire, as obtained from Eq. (7) using the nondispersive SRF and the dispersive SRF, respectively. Again, the nondispersive SRF (panel b, green filled curves) erroneously predicts a continuous redshift of the localized multipolar plasmon of order *m* in the nanowire with increasing *m*. On the other hand, the dispersive SRF (panel c, red filled curves) correctly reproduces the general behavior of plasmon resonances when compared to the TDDFT results (redshift with increasing *m* for low *m*, and blueshift for larger *m*). The results shown in Figure 4 thus confirm that the dispersive SRF can be used to model the nonlocal optical response of a cylindrical nanowire of small radius where the field localization and oscillation in space can be extreme.

#### 3.2.3 Individual spherical nanoparticles and nanoparticle dimers

We finally show that the dispersive SRF employed in this work can also be used to account for quantum surface effects and nonlocality in the optical response of plasmonic nanostructures with finite extension along the three spatial dimensions. As a first canonical plasmonic nanostructure, we consider a spherical Na metal nanoparticle (MNP) of radius *a* = 65.83 *a*_0_ (≈3.5nm) . On the one hand, this MNP size is sufficiently small so that we can perform TDDFT calculations as a reference and, on the other hand; it is large enough to ensure well-developed plasmon modes [104, 105]. Due to the small size of the nanoparticle, quantum effects and, in particular, nonlocality is expected to clearly affect the optical response [23, 30, 70, 75, 106]. Individual spherical MNPs support LMP resonances characterized by the angular momentum *ℓ*. The ratio *ℓ*(*ℓ* + 1)/*a* ^2^ in spherical MNPs when solving Laplace equation for the induced potential plays a similar role as $m^{2}/R_{c}^{2}$ in cylindrical nanowires and $k_{\|}^{2}$ in the slab geometry. Thus, we assign the effective wavenumber $k_{\|}^{\ell }=\sqrt{\ell \left(\ell +1\right)}/a$ along the transverse direction for spherical MNPs.

The spectrum of LMPs in spherical MNPS is determined by the nonretarded multipolar polarizability *α*_*ℓ*_(*ω*), which is given within the SRF by [56, 70]

(8) $$\alpha_{\ell }\left(\omega \right)\propto \frac{\left(\varepsilon \left(\omega \right)-1\right)\left(1+\frac{\ell }{a}d_{⊥}\left(\omega ,k_{\|}=k_{\|}^{\ell }\right)\right)}{\varepsilon \left(\omega \right)+\frac{\ell +1}{\ell }-\left(\varepsilon \left(\omega \right)-1\right)\frac{\ell +1}{a}d_{⊥}\left(\omega ,k_{\|}=k_{\|}^{\ell }\right)}.$$

**Figure 5:**
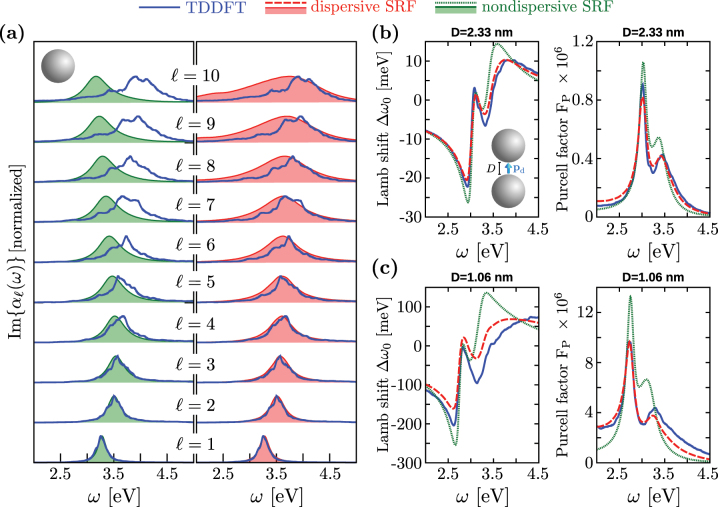
Comparison between the TDDFT (blue), dispersive SRF (red), and nondispersive SRF (green) results of the optical properties of individual spherical MNPs and dimers. (a) Imaginary part of the first ten multipolar polarizabilities *α*_*ℓ*_(*ω*) (*ℓ* = 1 − 10) of an individual spherical MNP. The left-hand side panel shows the comparison between TDDFT (blue lines) and nondispersive SRF (green filled curves) results, whereas the right-hand side panel shows the comparison between TDDFT and dispersive SRF (red filled curves) results. Each spectrum of Im{*α*_*ℓ*_(*ω*)} is normalized to 1 at its maximum, and vertically offset for clarity. (b, c) Lamb shift Δ*ω*_0_ (left-hand side panels) and Purcell factor F_P_ (right-hand side panels) obtained for a point-dipole emitter located at the center of a dimer of spherical MNPs of radius *a* = 65.83 *a*_0_ (≈3.5nm) . The dipole is oriented along the dimer axis, and its transition dipole moment is *μ* = 0.1 *e* nm (with *e* the electron charge). In (b), the gap separation is *D* = 2.33 nm. In (c), *D* = 1.06 nm.

We compare in Figure 5a the imaginary part of *α*_*ℓ*_(*ω*), Im{*α*_*ℓ*_(*ω*)}, obtained for different values of *ℓ* from TDDFT (blue lines), nondispersive SRF (left, green filled curves) and dispersive SRF (right, red filled curves). Details on the TDDFT calculations are given in ref. [75]. The LMPs of order *ℓ* appear as resonances in Im{*α*_*ℓ*_} associated with an increased absorption.

The TDDFT results of Im{*α*_*ℓ*_(*ω*)} in Figure 5a show that the LMP of order *ℓ* continuously blueshifts with increasing *ℓ* in the considered range *ℓ* = 1 − 10. The resonance broadens as *ℓ* increases due to the enhancement of surface-enabled Landau damping. In this situation, the coupling of the plasmon to electron–hole pair excitations and quantum finite-size effects lead to the emergence of discrete spectral features [42, 62] (notice that these features merge into a smooth resonant profile in the metallic nanowires studied above because of their larger radius). As already stated in ref. [75], the nondispersive SRF accurately reproduces the TDDFT results for low values of *ℓ* ∼ 1 − 4, but it cannot describe the correct behavior for larger angular momenta. Indeed, within the nondispersive SRF the plasmon resonances start to redshift with increasing *ℓ* for *ℓ* ≥ 5 (left-hand side panel in Figure 5a). On the other hand, when accounting for the dependence of the Feibelman parameter *d*_⊥_ on the effective wavenumber $k_{\|}^{\ell }=\sqrt{\ell \left(\ell +1\right)}/a$ , the dispersive SRF correctly captures the frequency blueshift of LMP resonances in Im{*α*_*ℓ*_(*ω*)} (right-hand side panel in Figure 5a). Although slight quantitative differences are present for large multipolar order *ℓ* = 7 − 10 (partially linked to the non-Lorentzian shape of Im{*α*_*ℓ*_(*ω*)} within TDDFT due to finite-size effects), qualitatively there is a good agreement between TDDFT and the dispersive SRF results over the entire range of *ℓ*-s considered here, and the improvement over the nondispersive SFR is evident.

Finally, we address another canonical plasmonic system: a dimer of spherical MNPs forming a plasmonic gap. Specifically, we study the case of a point-dipole emitter located at the center of the gap of size *D* formed by two identical spherical MNPs of radius *a* = 65.83 *a*_0_ (≈3.5nm) . The emitter is oriented along the axis of the MNPs dimer (*z*-axis). The gap separation distance, *D*, is in the nanometer scale, and thus a strong impact of the nonlocality on the optical response of the system is expected. We obtain the decay-rate enhancement (Purcell factor) and the change of the resonant frequency (Lamb shift) of the emitter due to its interaction with the MNPs dimer by calculating the imaginary and real parts of the electric field in the middle of the gap in response to the excitation of the point-dipole emitter [107]. The TDDFT results are obtained from the time evolution of the electron density in response to an impulsive potential created by a point dipole. The SRF results are obtained from the solution of Laplace’s equation by considering the electromagnetic coupling between different multipoles of the two individual MNPs. For the dispersive model we assign again $k_{\|}^{\ell }=\sqrt{\ell \left(\ell +1\right)}/a$ . Further details on the TDDFT and SRF calculations are provided in ref. [75]. The Lamb shift is calculated considering a transition dipole moment *μ* = 0.1 *e* nm (with *e* the electron charge).

Figure 5b shows the Lamb shift Δ*ω*_0_ (left) and Purcell factor *F*_P_ (right) obtained for a gap separation *D* = 2.33 nm, as calculated within the three models tested here (TDDFT, dispersive SRF, and nondispersive SRF). The three approximations show qualitatively good agreement. For this relatively large gap, the excitation of low-*ℓ* LMP resonances dominates the response of the MNPs dimer [75], validating the long-wavelength approximation behind the nondispersive SRF results for *D* = 2.33 nm. Note, nonetheless, that the results obtained within the dispersive SRF are more accurate when comparing to those obtained within TDDFT.

The significant improvement introduced by the dispersive SRF to describe the emitter–dimer electromagnetic interaction is more evident when considering a smaller gap, where field localization and thus higher multipolar activation occurs. Figure 5c shows the Lamb shift and Purcell factor obtained for a gap separation *D* = 1.06 nm. In this situation, because of the larger spatial confinement of the charges induced at the metallic surfaces across the gap, plasmon modes with high multipolar order *ℓ* become important. These high-*ℓ* modes show overlapping resonant frequencies and thus contribute to a single broad peak (referred to as pseudomode [108, 109]) at *ω* ∼ 3.4 eV as revealed by the TDDFT calculations (blue lines). Since the nondispersive model does not describe accurately high-*ℓ* multipolar modes for the individual MNP (Figure 5a), it clearly fails to reproduce the frequency and the width of the plasmon pseudomode obtained within TDDFT. As a consequence, the nondispersive SRF strongly overestimates the overlap between the pseudomode and the bonding dipolar plasmon resonance at *ω* ∼ 2.75 − 3 eV (green dotted lines), as observed in the Purcell factor and the Lamb shift. On the other hand, the dispersive SRF (red dashed lines) provides accurate results even for this small gap separation, further illustrating its ability to account for nonlocality in situations where plasmon-induced charges characterized by a rapid variation in the direction parallel to the metal surface are excited.

## 4 Summary and conclusions

We have introduced a *dispersive* surface-response formalism (SRF) that incorporates quantum surface effects and nonlocality into the optical response of plasmonic nanostructures via the so-called Feibelman parameters. While the *nondispersive* SRF typically implemented in the literature is based on the Feibelman parameter *d*_⊥_(*ω*) obtained within the long-wavelength limit (*k*_‖_ = 0), the dispersive SRF proposed here is based on a dispersive Feibelman parameter *d*_⊥_(*ω*, *k*_‖_) that explicitly depends on the wavenumber parallel to the metal surface, *k*_‖_. We obtain the values of *d*_⊥_(*ω*, *k*_‖_) from quantum many-body calculations for a planar metal–vacuum interface, and use a recent formulation of the electromagnetic boundary conditions [59] to account for the nonlocality of the dynamical screening in the direction parallel to the metal surface in various nanostructures.

Using TDDFT calculations as a reference, we have demonstrated that the *dispersive* SRF is significantly more accurate than the *nondispersive* SRF in describing plasmonic systems characterized by extremely confined induced fields. We have first shown that the *dispersive* SRF correctly describes the nonlocal dynamical screening of a planar metal surface and provides with the correct surface plasmon frequency dispersion with *k*_‖_. Further, we have demonstrated that the Feibelman parameter *d*_⊥_(*ω*, *k*_‖_) calculated in this work using a planar metal surface can also be used to address the nonlocal optical response of nonplanar nanostructures relevant in plasmonics. As examples, we have considered infinite cylindrical nanowires, spherical metallic nanoparticles, and nanoparticle dimers forming gaps described within the free-electron metal approximation. The symmetry of these nanostructures allowed us to introduce geometry-dependent effective parallel wavenumbers, $k_{\|}^{m}$ and $k_{\|}^{\ell }$ , in order to efficiently implement the *dispersive* SRF.

In all these systems, the *dispersive* SRF reproduces the TDDFT results that naturally incorporate quantum effects such as nonlocal dynamical screening, surface-enabled Landau damping, and the finite spatial extension of the induced electron density at the metal surface. This work thus establishes a milestone for the development of a theoretical model that captures quantum surface effects and nonlocality in extreme situations of field localization, while keeping the numerical efficiency and easy implementation of classical (local) electromagnetic theories [59, 110, 111]. Among others, our findings can be important for describing the optical response of metallic nanostructures interacting with fast electrons, nanoantennas coupled to molecules in close proximity, metallic nanoparticles with geometrical features characterized by small radii of curvature (such as picocavities), or nanoparticle ensembles with narrow gaps, all of them relevant situations in practical configurations of nowadays Nanophotonics.

## Supplementary Material

Supplementary Material Details
