# Supplementary material for: Dispersive surface-response formalism to address nonlocality in extreme plasmonic field confinement
Source: Nanophotonics. 2023 Jun 21;12(16):3277–89. doi: 10.1515/nanoph-2023-0178 (PMC11501702; doi:10.1515/nanoph-2023-0178)
Supplement: Supplementary file 1 — Supplementary Material Details [file j_nanoph-2023-0178_suppl_001.pdf]

Supplementary Material for: Dispersive surface-response formalism to address nonlocality in extreme plasmonic field confinement

Antton Babaze[1], Tomáš Neuman, Ruben Esteban, Javier Aizpurua[2], and Andrei G. Borisov[3]

[1]anttonbabaze@dipc.org; [2]aizpurua@ehu.eus; [3]andrei.borissov@universite-paris-saclay.fr

Contents

S1 Linear-response frequency-domain TDDFT calculations for a planar free-electron metal slab **S3**

S1.1 Ground-state calculations S3

S1.2 Linear-response calculations S4

S1.3 Reflection coefficient $R(\omega, k_{\|})$, surface response function $g(\omega, k_{\|})$, and Feibelman parameter $d_{\perp}(\omega, k_{\|})$ S7

S2 Real-time TDDFT calculations of the optical response of a cylindrical nanowire **S8**

S2.1 Ground-state calculations S9

S2.2 Optical response S10

S3 Surface-response formalism (SRF) for the optical response of a cylindrical nanowire **S11**

S3.1 Induced potential and the Feibelman parameter S11

S3.2 Plasmon resonances sustained by a cylindrical nanowire within the SRF S13

S4 Multipolar polarizabilities of a cylindrical nanowire **S15**

S4.1 General expressions of the multipolar polarizability and multipole moment S15

S4.2 Multipolar polarizability within the SRF and the classical theory S16

S4.3 Multipolar polarizability from TDDFT calculations S16

S5 Assessing the robustness of the dispersive Feibelman parameter **S18**

S5.1 Real-time TDDFT calculation of $d_{\perp}^{\mathrm{cyl}}(\omega, m)$ S18

S5.2 Comparison between the dispersive Feibelman parameter $d_{\perp}^{\mathrm{cyl}}(\omega, m)$ calculated with TDDFT for different nanowire sizes and $d_{\perp}(\omega, k_{\|})$ calculated with TDLDA for the planar metal surface. S20

S6 Resonant frequency and width of the plasmon resonances **S22**

Atomic units (a.u.) are used throughout this supplementary material unless otherwise stated.

S1 Linear-response frequency-domain TDDFT calculations for a planar free-electron metal slab

To calculate the physical quantities relevant for the analysis of the surface plasmons and for the dispersive surface response formalism (SRF) proposed in the main text, we employ a many-body approach that allows for obtaining the nonlocal linear optical response of a planar metal slab to a given external potential $V_{\text{ext}}(\mathbf{r}, \omega)$. This many-body approach is based on the linear-response frequency-domain time-dependent density functional theory (TDDFT). We provide below the details on the ground-state calculations, the linear-response calculations, and the calculation of the reflection coefficient, surface response function and the Feibelman parameter $d_\perp(\omega, k_\|)$.

S1.1 Ground-state calculations

To model the response of the metal surface, we consider the slab geometry depicted in Fig. 1a of the main text. The slab is infinite in the (x, y)-plane and has a thickness L along the z-axis perpendicular to the metal–vacuum interfaces. We set the position $z = 0$ at the upper metal–vacuum interface ($z = -L$ corresponds to the lower metal surface), and L is set large enough to avoid the interaction between the two opposite metal–vacuum interfaces for the values of $k_\|$ considered. The electronic structure of the slab is described within the jellium model of free-electron metals [S1, S2, S3, S4, S5] using a Wigner–Seitz radius $r_s = 4$ a$_0$ that corresponds to sodium (a$_0 = 0.0529$ nm is the Bohr radius).

The ground state of the metal slab is obtained here using the Kohn–Sham (KS) scheme of density-functional theory (DFT). Due to the translational invariance of the system in the (x, y)-plane, the ground-state single-particle wave functions (KS orbitals) can be expressed as follows:

$$\psi_i(\mathbf{q}; \mathbf{r}) = \frac{1}{2\pi} \varphi_i(z) e^{i\mathbf{q}\cdot\mathbf{r}_\|}, \tag{S1}$$

where $\mathbf{r} = (x, y, z)$ is the 3D position vector, $\mathbf{r}_\| = (x, y)$ is the position vector parallel to the interface, and $\mathbf{q}$ is the wavevector associated to the electron motion in the direction parallel to the surface. The one-dimensional wave functions $\varphi_i(z)$ are discrete self-consistent solutions of the KS equations

$$\left[-\frac{1}{2} \frac{d^2}{dz^2} + V_{\text{H}}[n_0(z)] + V_{\text{xc}}[n_0(z)] \right] \varphi_i(z) = \epsilon_i \varphi_i(z), \tag{S2}$$

with ϵ_i being the corresponding KS energies. Each ϵ_i defines the bottom of a parabolic band i of energy

$$E_i(\mathbf{q}) = \epsilon_i + |\mathbf{q}|^2/2. \tag{S3}$$

In Eq. (S2), $V_{\text{xc}}[n_0(z)]$ is the exchange and correlation potential, here described using the kernel of Gunnarsson and Lundqvist [S6] within the local-density approximation (LDA) [S7]. The Hartree potential, $V_{\text{H}}[n_0(z)]$, is obtained from the solution of Poisson's equation with the charge density $n_+(z) - n_0(z)$. The positive background density, $n_+(z)$, is nonzero only inside the slab, $n_+(z) = \left(\frac{4\pi}{3} r_s^3\right)^{-1}$ for $-L \leq z \leq 0$, and the ground-state electron density can be found from

$$n_0(z) = \sum_i \zeta_i |\varphi_i(z)|^2, \tag{S4}$$

where the sum runs over the occupied KS orbitals with energies $\epsilon_i \leq E_{\mathrm{F}}$ (E_{F} is the Fermi energy). The statistical factors ζ_i account for spin and $\mathbf{q}$ degeneracy, and it is given for this slab geometry by

$$\zeta_i = \frac{1}{\pi}\left(E_{\mathrm{F}} - \epsilon_i\right). \tag{S5}$$

S1.2 Linear-response calculations

To obtain the optical response of the metal surface we employ linear-response frequency-domain TDDFT calculations. The methodology adopted here is based on the formalism described in ref. S7, referred to as the time-dependent local density approximation (TDLDA). In contrast to e.g. the widely employed random-phase approximation (RPA), TDLDA accounts for the variation of the exchange–correlation potential due to the variation of the electron density induced by an external potential. Within the TDLDA, the electron charge density $\rho_{\mathrm{ind}}(\mathbf{r},\omega)$ induced in the metal in response to an external potential $V_{\mathrm{ext}}(\mathbf{r},\omega)$ can be obtained as:

$$\rho_{\mathrm{ind}}(\mathbf{r},\omega) = -\delta n(\mathbf{r},\omega) = \int \mathrm{d}^3\mathbf{r}'\, \chi(\mathbf{r},\mathbf{r}',\omega) V_{\mathrm{ext}}(\mathbf{r}',\omega). \tag{S6}$$

Here, $\chi(\mathbf{r},\mathbf{r}',\omega)$ is the first-order *many-body response function* (or linear susceptibility) [S7] containing all the information about the optical response of the metal slab, and $\delta n(\mathbf{r},\omega)$ is the induced electron (number) density. Importantly, $\chi(\mathbf{r},\mathbf{r}',\omega)$ contains the information about the geometry of the metal slab and should therefore not be confused with the bulk susceptibility of the material.

In the framework of TDLDA, $\chi(\mathbf{r},\mathbf{r}',\omega)$ can be linked to a simpler *independent-electron response function* $\chi^0(\mathbf{r},\mathbf{r}',\omega)$ that expresses the response of the metallic system to the total effective or screened local potential $V_{\mathrm{tot}}(\mathbf{r},\omega) = V_{\mathrm{ext}}(\mathbf{r},\omega) + V_{\mathrm{ind}}(\mathbf{r},\omega)$. Here $V_{\mathrm{ind}}(\mathbf{r},\omega)$ is the induced potential created by the induced electron charge density:

$$\rho_{\mathrm{ind}}(\mathbf{r},\omega) = \int \mathrm{d}^3\mathbf{r}'\, \chi^0(\mathbf{r},\mathbf{r}',\omega) V_{\mathrm{tot}}(\mathbf{r}',\omega). \tag{S7}$$

Further, $V_{\mathrm{ind}}(\mathbf{r},\omega)$ can be explicitly expressed as:

$$V_{\mathrm{ind}}(\mathbf{r},\omega) = \int \mathrm{d}^3\mathbf{r}'\, v(\mathbf{r},\mathbf{r}')\rho_{\mathrm{ind}}(\mathbf{r}',\omega), \tag{S8}$$

where $v(\mathbf{r},\mathbf{r}') = v_{\mathrm{c}}(\mathbf{r},\mathbf{r}') + v_{\mathrm{xc}}(\mathbf{r},\mathbf{r}')$ is the kernel consisting of the Coulomb-interaction kernel, $v_{\mathrm{c}}(\mathbf{r},\mathbf{r}')$, and the exchange–correlation kernel, $v_{\mathrm{xc}}(\mathbf{r},\mathbf{r}')$. The latter is obtained within the LDA from the exchange–correlation potential $V_{\mathrm{xc}}[n(\mathbf{r})]$ of Gunnarsson and Lundqvist as [S6]

$$v_{\mathrm{xc}}(\mathbf{r},\mathbf{r}',\omega) = \left.\frac{\delta V_{\mathrm{xc}}[n(\mathbf{r})]}{\delta n(\mathbf{r})}\right|_{n(\mathbf{r})=n_0(\mathbf{r})} \delta(\mathbf{r}-\mathbf{r}'). \tag{S9}$$

Equations (S6)–(S8) can be combined to yield a self-consistent equation for the linear susceptibility $\chi(\mathbf{r},\mathbf{r}',\omega)$:

$$\begin{aligned}\chi(\mathbf{r},\mathbf{r}',\omega) &= \chi^0(\mathbf{r},\mathbf{r}',\omega)\\ &+ \int \mathrm{d}^3\mathbf{r}''\,\mathrm{d}^3\mathbf{r}'''\, \chi^0(\mathbf{r},\mathbf{r}''',\omega) v(\mathbf{r}''',\mathbf{r}'')\chi(\mathbf{r}'',\mathbf{r}',\omega).\end{aligned} \tag{S10}$$

Within the TDLDA, $\chi^0(\mathbf{r}, \mathbf{r}', \omega)$ can be expressed using the noninteracting single-particle KS orbitals and energies as:

$$\chi^0(\mathbf{r}, \mathbf{r}', \omega) = 2 \sum_{ij} \iint \mathrm{d}^2\mathbf{q}' \mathrm{d}^2\mathbf{q}'' (f_i(\mathbf{q}') - f_j(\mathbf{q}'')) \frac{\psi_i^*(\mathbf{q}'; \mathbf{r}) \psi_i(\mathbf{q}'; \mathbf{r}') \psi_j^*(\mathbf{q}''; \mathbf{r}) \psi_j(\mathbf{q}''; \mathbf{r}')}{E_i(\mathbf{q}') - E_j(\mathbf{q}'') + \omega + i\gamma}, \quad \text{(S11)}$$

where $\psi_i(\mathbf{q}; \mathbf{r})$ are the ground-state KS orbitals (Eq. (S1)) and $E_i(\mathbf{q})$ the corresponding energy band (Eq. (S3)). In Eq. (S11), $\gamma = \eta/2 = 75$ meV is a phenomenological damping parameter set to be consistent with the attenuation introduced in the TDDFT calculations discussed below, and the factor 2 accounts for spin. For the zero-temperature case considered here, the occupation number of level i denoted as $f_i(\mathbf{q})$ in Eq. (S11) is given by

$$f_i(\mathbf{q}) = \begin{cases} 1, & \text{for} \quad E_i(\mathbf{q}) \leq E_\mathrm{F}, \quad \text{i.e.} \quad |\mathbf{q}|^2 < 2(E_\mathrm{F} - \epsilon_i) \equiv K_i^2 \\ 0, & \text{otherwise.} \end{cases} \quad \text{(S12)}$$

It is further convenient to express the linear susceptibility $\chi(\mathbf{r}, \mathbf{r}', \omega)$ in the form of its surface Fourier transform (with $\mathbf{k}_\parallel$ the wavevector parallel to the surface and $k_\parallel = |\mathbf{k}_\parallel|$):

$$\chi(\mathbf{r}, \mathbf{r}', \omega) = \int \frac{\mathrm{d}^2\mathbf{k}_\parallel}{(2\pi)^2} \, \tilde{\chi}(z, z', \omega, \mathbf{k}_\parallel) \, e^{i\mathbf{k}_\parallel \cdot (\mathbf{r}_\parallel - \mathbf{r}'_\parallel)}, \quad \text{(S13)}$$

where $\tilde{\chi}(z, z', \omega, \mathbf{k}_\parallel)$ is the linear susceptibility in $\mathbf{k}_\parallel$-space.

The self-consistent equation given by Eq. (S10) remains valid when we transform all the quantities to the Fourier space:

$$\begin{aligned} \tilde{\chi}(z, z', \omega, \mathbf{k}_\parallel) &= \tilde{\chi}^0(z, z', \omega, \mathbf{k}_\parallel) \\ &+ \int \mathrm{d}z'' \, \mathrm{d}z''' \, \tilde{\chi}^0(z, z''', \omega, \mathbf{k}_\parallel) \tilde{v}(z''', z'', \mathbf{k}_\parallel) \tilde{\chi}(z'', z', \omega, \mathbf{k}_\parallel), \end{aligned} \quad \text{(S14)}$$

with

$$\tilde{v}(z, z', \mathbf{k}_\parallel) = \tilde{v}_\mathrm{c}(z, z', \mathbf{k}_\parallel) + f_\mathrm{xc}(z) \delta(z - z'). \quad \text{(S15)}$$

In Eq. (S15), $\delta(z - z')$ is the Dirac delta function, $\tilde{v}_\mathrm{c}(z, z', \mathbf{k}_\parallel)$ is the Fourier component of the Coulomb-interaction kernel given by

$$\tilde{v}_\mathrm{c}(z, z', \mathbf{k}_\parallel) = \frac{2\pi}{k_\parallel} e^{-k_\parallel |z - z'|}, \quad \text{(S16)}$$

and

$$f_\mathrm{xc}(z) = \left[\frac{0.611}{r_s(z)^2} + \frac{0.3796}{(r_s(z) + 11.4) r_s(z)} \right] \left(\frac{3}{4\pi} \right)^{\frac{1}{3}} \left(-\frac{1}{3} \right) n_0(z)^{-\frac{4}{3}}, \quad \text{(S17)}$$

where $r_s(z)$ is the local Wigner–Seitz radius defined by the electron density $n_0(z)$ as

$$r_s(z) = \left(\frac{3}{4\pi n_0(z)} \right)^{\frac{1}{3}}. \quad \text{(S18)}$$

Notice that Eq. (S14) is valid for each value of $\mathbf{k}_\parallel$ and ω independently.

The Fourier components of the independent-electron response function $\tilde{\chi}^0(z, z', \omega, \mathbf{k}_\parallel)$ can be found from:

$$\tilde{\chi}^0(z, z', \omega, \mathbf{k}_\parallel) = 2\sum_{ij} X_{ij}(\omega, \mathbf{k}_\parallel)\varphi_i(z)\varphi_i^*(z')\varphi_j^*(z)\varphi_j(z'), \tag{S19}$$

where $\varphi_i(z)$ is obtained from Eq. (S2), the summation runs over both occupied and unoccupied bands i and j, and

$$X_{ij}(\omega, \mathbf{k}_\parallel) = \int \frac{\mathrm{d}^2\mathbf{q}}{(2\pi)^2} \frac{f_i(\mathbf{q} - \mathbf{k}_\parallel) - f_j(\mathbf{q})}{[E_i(\mathbf{q} - \mathbf{k}_\parallel) - E_j(\mathbf{q})] + \omega + i\gamma}. \tag{S20}$$

Without loss of generality, we choose φ_i to be real-valued functions. Further, the summation in Eq. (S19) can be rearranged so that

$$\tilde{\chi}^0(z, z', \omega, \mathbf{k}_\parallel) = 2\sum_{ij} X'_{ij}(\omega, \mathbf{k}_\parallel)\varphi_i(z)\varphi_i(z')\varphi_j(z)\varphi_j(z'), \tag{S21}$$

where j only runs over filled bands and i runs over all bands [this is in contrast to the original expression in Eq. (S19)]. The coefficient $X'_{ij}(\omega, \mathbf{k}_\parallel)$ results from the rearrangement of the summation and is given by:

$$X'_{ij}(\omega, \mathbf{k}_\parallel) = \int \frac{\mathrm{d}^2\mathbf{q}}{(2\pi)^2} \left[\frac{-f_j(\mathbf{q})}{[E_i(\mathbf{q} - \mathbf{k}_\parallel) - E_j(\mathbf{q})] - (\omega + i\gamma)} + \frac{-f_j(\mathbf{q})}{[E_i(\mathbf{q} + \mathbf{k}_\parallel) - E_j(\mathbf{q})] + (\omega + i\gamma)} \right]. \tag{S22}$$

We further transform the integral in Eq. (S22) into polar coordinates $\mathbf{q} = (q\cos\theta, q\sin\theta)$ using

$$\int \frac{\mathrm{d}^2\mathbf{q}}{(2\pi)^2} = \frac{1}{(2\pi)^2} \int_0^\infty \mathrm{d}q\, q \int_0^{2\pi} \mathrm{d}\theta. \tag{S23}$$

With the help of the result [S8]

$$\int_0^{2\pi} \mathrm{d}\theta \, \frac{1}{\beta \pm \alpha\cos\theta \pm i\tilde{\gamma}} = \mp \frac{2\pi i}{\sqrt{\alpha^2 - (\beta \pm i\tilde{\gamma})^2}}, \tag{S24}$$

where α, β, and $\tilde{\gamma}$ are constants, we perform the angular integral in Eq. (S22). For the zero-temperature case the occupation numbers $f_i(\mathbf{q})$ are given by Eq. (S12), and the integral can then be solved analytically, yielding:

$$\begin{aligned} X'_{ij}(\omega, \mathbf{k}_\parallel) &= \\ &= \frac{i}{2\pi k_\parallel^2} \Bigg\{ \left[\sqrt{k_\parallel^2 K_i^2 - (\beta_{ij}^- - i\gamma)^2} - i\sqrt{(\beta_{ij}^- - i\gamma)^2} \right] \\ &\qquad - \left[\sqrt{k_\parallel^2 K_i^2 - (\beta_{ij}^+ + i\gamma)^2} - i\sqrt{(\beta_{ij}^+ + i\gamma)^2} \right] \Bigg\}, \end{aligned} \tag{S25}$$

with

$$\beta_{ij}^{\pm} = \epsilon_i - \epsilon_j + \frac{k}{2} \pm \omega. \tag{S26}$$

In our numerical implementation, we discretize the response functions in space by transforming them into the orthonormal basis given by the following basis functions,

$$s_n(z) = \sqrt{\frac{2}{z_{\rm max} - z_{\rm min}}}\ \sin\left(n\pi\ \frac{z - z_{\rm min}}{z_{\rm max} - z_{\rm min}}\right), \tag{S27}$$

on the interval $z_{\rm min} \leq z \leq z_{\rm max}$. We choose the boundaries $z_{\rm min} = -L - \Lambda$ and $z_{\rm max} = \Lambda$ to be positioned at a distance Λ from the metal–vacuum interfaces outside the metal slab in the region where the electron density becomes negligible. In practice, we set $\Lambda = 34.5$ $\mathrm{a_0}$.

We can express all the quantities in this basis as follows. For a generic kernel $\mathcal{K}(z, z')$ we define:

$$[\underline{\underline{\mathcal{K}}}]_{mn} = \mathcal{K}_{mn} = \int_{z_{\rm min}}^{z_{\rm max}} \mathrm{d}z \int_{z_{\rm min}}^{z_{\rm max}} \mathrm{d}z' s_m(z)\mathcal{K}(z, z')s_n(z'). \tag{S28}$$

For completeness, the inverse transform is:

$$\mathcal{K}(z, z') = \sum_{mn} s_m(z)\mathcal{K}_{mn}s_n(z'). \tag{S29}$$

A generic function $F(z)$ of the z coordinate transforms as

$$[\underline{F}]_n = F_n = \int_{z_{\rm min}}^{z_{\rm max}} dz\ s_n(z)F(z), \tag{S30}$$

and

$$F(z) = \sum_n F_n s_n(z). \tag{S31}$$

We rewrite the self-consistent equation given by Eq. (S14) as a matrix equation

$$\underline{\underline{\tilde{\chi}}}(\omega, \mathbf{k}_\parallel) = \underline{\underline{\tilde{\chi}}}^0(\omega, \mathbf{k}_\parallel) + \underline{\underline{\tilde{\chi}}}^0(\omega, \mathbf{k}_\parallel) \cdot \underline{\underline{\tilde{v}}}(\omega, \mathbf{k}_\parallel) \cdot \underline{\underline{\tilde{\chi}}}(\omega, \mathbf{k}_\parallel), \tag{S32}$$

from which we readily obtain $\underline{\underline{\tilde{\chi}}}(\omega, \mathbf{k}_\parallel)$ as:

$$\underline{\underline{\tilde{\chi}}}(\omega, \mathbf{k}_\parallel) = \left[\underline{\underline{I}} - \underline{\underline{\tilde{\chi}}}^0(\omega, \mathbf{k}_\parallel) \cdot \underline{\underline{\tilde{v}}}(\omega, \mathbf{k}_\parallel)\right]^{-1} \cdot \underline{\underline{\tilde{\chi}}}^0(\omega, \mathbf{k}_\parallel), \tag{S33}$$

with $\underline{\underline{I}}$ the identity matrix.

S1.3 Reflection coefficient $R(\omega, k_\parallel)$, surface response function $g(\omega, k_\parallel)$, and Feibelman parameter $d_\perp(\omega, k_\parallel)$

We next use the response function $\underline{\underline{\tilde{\chi}}}(\omega, \mathbf{k}_\parallel)$ given by Eq. (S33) to derive the reflection coefficient $R(\omega, \mathbf{k}_\parallel)$. For a Fourier component of the external potential $\tilde{V}_{\rm ext}(z, \omega, \mathbf{k}_\parallel) = \frac{2\pi}{k_\parallel} e^{k_\parallel z}$ exponentially

decaying into the slab, the reflection coefficient $R(\omega, \mathbf{k}_{||})$ determines the amplitude of the Fourier component of the induced potential $\tilde{V}_{\text{ind}}(z, \omega, \mathbf{k}_{||})$ at position z in vacuum, where

$$\tilde{V}_{\text{ind}}(z, \omega, \mathbf{k}_{||}) = \frac{2\pi}{k_{||}} R(\omega, \mathbf{k}_{||}) e^{-k_{||} z}. \tag{S34}$$

The induced potential is obtained from

$$\tilde{V}_{\text{ind}}(z, \omega, \mathbf{k}_{||}) = \underline{\tilde{v}_{\text{c}}}(z, \mathbf{k}_{||}) \cdot \underline{\underline{\tilde{\chi}}}(\omega, \mathbf{k}_{||}) \cdot \underline{\tilde{V}_{\text{ext}}}(z, \omega, \mathbf{k}_{||}), \tag{S35}$$

where $\underline{\tilde{v}_{\text{c}}}(z, \mathbf{k}_{||})$ is the Coulomb-interaction kernel partially transformed into the sine basis (it becomes a row vector):

$$[\underline{\tilde{v}_{\text{c}}}(z, \mathbf{k}_{||})]_n = \int \mathrm{d}z'\, s_n(z') \tilde{v}_{\text{c}}(z, z', \mathbf{k}_{||}). \tag{S36}$$

We remark that the reflection coefficient can be defined with the opposite sign to correspond to the Fresnel coefficient R^{p} for p-polarized electric field $R^{\text{p}}(\omega, \mathbf{k}_{||}) = -R(\omega, \mathbf{k}_{||})$. This stems from the fact that the electric field of the incident and reflected exponential waves defined here point in opposing directions.

With the present choice of the external potential, $\tilde{V}_{\text{ext}}(z, \omega, \mathbf{k}_{||}) = \frac{2\pi}{k_{||}} e^{k_{||} z}$, the surface response function $g(\omega, \mathbf{k}_{||})$ equals to the reflection coefficient defined above, $g(\omega, \mathbf{k}_{||}) = R(\omega, \mathbf{k}_{||})$. For a rigorous demonstration, we address the reader to an excellent discussion in ref. S7. We obtain the dispersion relationship and widths of propagating plasmons at a sodium planar surface by analyzing the resonances in the surface loss function, which is given by the imaginary part of $g(\omega, \mathbf{k}_{||})$.

Finally, we also use the TDLDA formalism to calculate the Feibelman parameter $d_\perp(\omega, \mathbf{k}_{||})$. The Feibelman parameter is defined for the metal–vacuum interface at $z = 0$. Using the external potential $\tilde{V}_{\text{ext}}(z, \omega, \mathbf{k}_{||}) = \frac{2\pi}{k_{||}} e^{k_{||} z}$, we obtain the induced electron density $\delta\tilde{n}(z, \omega, \mathbf{k}_{||}) = -\tilde{\rho}_{\text{ind}}(z, \omega, \mathbf{k}_{||}) = -\underline{\tilde{\chi}}(z, \omega, \mathbf{k}_{||}) \cdot \underline{\tilde{V}_{\text{ext}}}(z, \omega, \mathbf{k}_{||})$, with

$$[\underline{\tilde{\chi}}(z, \omega, \mathbf{k}_{||})]_n = \int \mathrm{d}z'\, s_n(z') \tilde{\chi}(z, z', \omega, \mathbf{k}_{||}), \tag{S37}$$

and extract the Feibelman parameter $d_\perp(\omega, \mathbf{k}_{||})$ as:

$$d_\perp(\omega, \mathbf{k}_{||}) = \frac{\int z \delta\tilde{n}(z, \omega, \mathbf{k}_{||})\, \mathrm{d}z}{\int \delta\tilde{n}(z, \omega, \mathbf{k}_{||})\, \mathrm{d}z}. \tag{S38}$$

To simplify the notation, in the main text we use $d_\perp(\omega, k_{||})$, $\delta n(z, \omega, k_{||})$, $g(\omega, k_{||})$, $V_{\text{ext}}(z, \omega, k_{||})$, and $\chi(z, z', \omega, k_{||})$ to refer to $d_\perp(\omega, \mathbf{k}_{||})$, $\delta\tilde{n}(z, \omega, \mathbf{k}_{||})$, $g(\omega, \mathbf{k}_{||})$, $\tilde{V}_{\text{ext}}(z, \omega, \mathbf{k}_{||})$ and $\tilde{\chi}(z, z', \omega, \mathbf{k}_{||})$, respectively.

S2 Real-time TDDFT calculations of the optical response of a cylindrical nanowire

In this section, we explain the procedure adopted within real-time TDDFT to obtain the optical response of a cylindrical nanowire of radius R_c. The electronic structure of the nanowire is

described within the same jellium model of free-electron metals as the one used for the metal slab above. We consider sodium metal with Wigner–Seitz radius $r_s = 4$ a$_0$. The nanowire is infinite along the z-axis, and thus it is translationally invariant with respect to the z-axis (see the geometry of the system in Fig. 1b of the main text). The nanowire is also rotationally invariant with respect to the azimuth angle φ. The radius R_c of the nanowire is given by

$$R_c = \sqrt{\frac{4}{3} N_e \, r_s^3}, \tag{S39}$$

where N_e is the number of electrons per unit length in the z-direction considered in the simulations. In this work, we consider $N_e = 65.9$ a$_0^{-1}$, 117.2 a$_0^{-1}$ and 263.7 a$_0^{-1}$ leading to $R_c = 75$ a$_0$, 100 a$_0$ and 150 a$_0$, respectively ($a_0 = 0.0529$ nm is the Bohr radius).

S2.1 Ground-state calculations

The ground state of the system is obtained using the KS scheme of DFT. Due to the translational invariance and cylindrical symmetry with respect to the z-axis, the ground-state KS orbitals $\Omega^0_{j_\rho,m,q_z}(\mathbf{r})$ are represented in cylindrical coordinates $\mathbf{r} = \{\rho, \varphi, z\}$ and expressed as

$$\Omega^0_{j_\rho,m,q_z}(\mathbf{r}) = \psi^0_{j_\rho,m}(\rho) \frac{1}{\sqrt{2\pi}} e^{im\varphi} \, \frac{1}{\sqrt{2\pi}} e^{iq_z z}, \tag{S40}$$

where m is the magnetic quantum number, and $\psi^0_{j_\rho,m}(\rho)$ is the radial part of the KS orbitals with radial quantum number j_ρ. The radial part $\psi^0_{j_\rho,m}(\rho)$ is obtained by solving static KS equations in 2D describing the electron motion in the (x, y)-plane,

$$\hat{H}_0[n_0(\rho,\varphi)]\psi^0_{j_\rho,m}(\rho,\varphi) = \epsilon_{j_\rho,m}\psi^0_{j_\rho,m}(\rho,\varphi), \tag{S41}$$

where $\epsilon_{j_\rho,m}$ are the KS eigenenergies of the non-interacting single-electron system. The static Hamiltonian $H_0[n_0(\rho,\varphi)]$ is given by

$$\hat{H}_0[n_0(\rho,\varphi)] = \hat{T}_\rho + V_{\mathrm{H}}[n_0(\rho,\varphi)] + V_{\mathrm{xc}}[n_0(\rho,\varphi)]. \tag{S42}$$

In Eq. (S42),

$$\hat{T}_\rho = -\frac{1}{2}\left(\frac{1}{\rho}\frac{\partial}{\partial\rho}\left(\rho\frac{\partial}{\partial\rho}\right) - \frac{m^2}{\rho^2}\right) \tag{S43}$$

is the kinetic-energy operator.

The exchange and correlation potential, $V_{\mathrm{xc}}[n_0(\rho,\varphi)]$, is described using the kernel of Gunnarsson and Lundqvist [S6] within the LDA. The Hartree potential, $V_{\mathrm{H}}[n_0(\rho,\varphi)]$, is obtained from the solution of Poisson's equation, i.e., retardation effects are neglected due to the small relevant size of the system. Finally, the equilibrium electron density $n_0(\rho,\varphi)$ is given by

$$n_0(\rho,\varphi) \equiv n_0(\rho) = \sum_{(j_\rho,m)\subset occ} \zeta_{j_\rho,m}|\psi^0_{j_\rho,m}(\rho)|^2, \tag{S44}$$

where the sum runs over the occupied (occ) orbitals with energies $\epsilon_{j_\rho,m} \leq E_{\mathrm{F}}$ (E_{F} is the Fermi energy), and the statistical factors $\zeta_{j_\rho,m}$ account for spin degeneracy as well as for the contribution of different q_z-states associated with the electron motion along the z-axis. For the cylindrical nanowire, $\zeta_{j_\rho,m}$ is given by

$$\zeta_{j_\rho,m} = \frac{1}{\pi^2}\sqrt{2(E_{\mathrm{F}} - \epsilon_{j_\rho,m})}. \tag{S45}$$

S2.2 Optical response

To obtain the optical response of the cylindrical nanowire, we use real-time TDDFT calculations using the methodology employed in prior works [S9, S10, S11, S12, S13]. Since we are interested in the situation where the external potential does not depend on the z-coordinate, the z-dependence of the KS orbitals factorizes out similarly to the ground-state calculations. The time-dependent KS orbitals of the system are then sought in Cartesian coordinates in the form

$$\Omega_{j,q_z}(\mathbf{r},t) = \psi_j(x,y,t)\ \frac{1}{\sqrt{2\pi}} e^{iq_z z}. \tag{S46}$$

The wave functions $\psi_j(x,y,t)$ evolve in time according to the 2D time-dependent KS equations,

$$i\frac{\partial}{\partial t}\psi_j(x,y,t) = \left[\hat{T} + V_H[n(x,y,t)] + V_{xc}[n(x,y,t)] + V_{\text{ext}}(x,y,t)\right]\ \psi_j(x,y,t), \tag{S47}$$

where $\hat{T} = -\frac{1}{2}\left(\frac{\partial^2}{\partial x^2} + \frac{\partial^2}{\partial y^2}\right)$ is the kinetic-energy operator, $V_H[n(x,y,t)]$ is the Hartree potential and $V_{xc}[n(x,y,t)]$ is the exchange–correlation potential. The Hartree and exchange–correlation potentials depend on time through the time dependence of the electron density $n(x,y,t)$. For $V_{xc}[n(x,y,t)]$, we use the adiabatic local-density approximation (ALDA) with the exchange–correlation kernel of Gunnarsson and Lundqvist [S6] consistent with static DFT calculations. Finally, $V_{\text{ext}}(x,y,t)$ is the external potential acting on the system and triggering its time evolution.

The initial conditions $\psi_j(x,y,t=0)$ are given by the *occupied* KS orbitals of the ground state,

$$\psi_j(x,y,t=0) = \psi^0_{j_\rho,m}(\rho)\frac{1}{\sqrt{2\pi}} e^{im\varphi}, \tag{S48}$$

where $\rho = \sqrt{x^2+y^2}$, and the azimuth angle φ satisfies the relations $x = \rho\cos(\varphi)$, and $y = \rho\sin(\varphi)$. Note that the quantum number j enumerates the variation of both j_ρ and m, where $\{j_\rho, m\} \subset occ$. The time-dependent electron density of the system, $n(x,y,t)$, is obtained from

$$n(x,y,t) = \sum_j \zeta_j \left|\psi_j(x,y,t)\right|^2, \tag{S49}$$

where the statistical factors ζ_j are defined as

$$\zeta_j = \frac{2}{\pi}\sqrt{2(E_{\text{F}} - \epsilon_j)}, \tag{S50}$$

and ϵ_j is given by the corresponding $\epsilon_{j_\rho,m}$.

The KS equations given by Eqs. (S47)–(S49) are solved self-consistently in real time using the short-time propagation (time-step $\Delta t = 0.1$ a.u.) and split-operator technique [S14, S15, S16]. The time-dependent KS orbitals $\psi_j(x,y,t)$ are represented on an equidistant mesh in Cartesian coordinates with mesh step $\Delta x = \Delta y = 0.48$ a$_0$. The pseudospectral Fourier-grid method [S17, S18] is then employed for an efficient calculation of the kinetic-energy operator as well as for the calculation of the Hartree potential V_{H} from Poisson's equation.

S3 Surface-response formalism (SRF) for the optical response of a cylindrical nanowire

S3.1 Induced potential and the Feibelman parameter

Consider a cylindrical nanowire of radius R_c infinite along the z-axis and subjected to a harmonic external perturbation. The screening of the external potential is associated with the electron density $\delta n(\mathbf{r}, \omega)$ induced in the cylinder and with the corresponding charge density $\rho_{\text{ind}}(\mathbf{r}, \omega) = -\delta n(\mathbf{r}, \omega)$. In this section, and consistent with the previous section on TDDFT calculations, we assume that the external perturbation is independent of z, i.e., the system keeps translational invariance along the z-axis. Using cylindrical coordinates, the induced potential $\phi(\rho, \varphi, \omega)$ can be then found from Poisson's equation, $\nabla^2\phi(\rho, \varphi, \omega) = 4\pi\delta n(\rho, \varphi, \omega)$, which is cast in the form

$$\left[\frac{1}{\rho}\frac{\partial}{\partial\rho}\left(\rho\frac{\partial}{\partial\rho}\right) - \frac{m^2}{\rho^2}\right] \phi_m(\rho, \omega) = 4\pi\delta n_m(\rho, \omega), \tag{S51}$$

where $\phi_m(\rho, \omega)$ are the moments of the induced potential

$$\phi(\rho, \varphi, \omega) = \sum_m \phi_m(\rho, \omega)\, e^{im\varphi}, \tag{S52}$$

and $\delta n_m(\rho, \omega)$ are the moments of the induced electron density $\delta n(\rho, \varphi, \omega)$

$$\delta n(\rho, \varphi, \omega) = \sum_m \delta n_m(\rho, \omega)\, e^{im\varphi}. \tag{S53}$$

To solve Eq. (S51), we first find the Green's function $G_m(\rho, \rho')$ that satisfies the following equation:

$$\left[\frac{1}{\rho}\frac{\partial}{\partial\rho}\left(\rho\frac{\partial}{\partial\rho}\right) - \frac{m^2}{\rho^2}\right] G_m(\rho, \rho') = \frac{1}{\rho}\delta(\rho - \rho'), \tag{S54}$$

so that

$$\phi_m(\rho, \omega) = 4\pi \int_0^\infty \rho' d\rho' G_m(\rho, \rho')\delta n_m(\rho', \omega). \tag{S55}$$

The solution of Eq. (S54) with proper boundary conditions and for $m \neq 0$ is given by

$$G_m(\rho, \rho') = \begin{cases} -\frac{1}{2|m|}\left(\frac{\rho}{\rho'}\right)^{|m|}, & \text{for } \rho < \rho' \\ \\ -\frac{1}{2|m|}\left(\frac{\rho'}{\rho}\right)^{|m|}, & \text{for } \rho > \rho' \end{cases}. \tag{S56}$$

Thus, according to Eq. (S55), the radial part of the induced potential $\phi_m(\rho, \omega)$ (see Eq. (S52)) is given by

$$\phi_m(\rho, \omega) = -2\frac{\pi}{|m|}\left[\int_0^\rho \rho'\, d\rho' \left(\frac{\rho'}{\rho}\right)^{|m|} \delta n_m(\rho', \omega) + \int_\rho^\infty \rho'\, d\rho' \left(\frac{\rho}{\rho'}\right)^{|m|} \delta n_m(\rho', \omega)\right]. \tag{S57}$$

For example, within the classical local theory, the induced electron density is strictly located at the jellium edge of the nanowire, i.e., $n_m(\rho, \omega) = \sigma_m(\omega)\delta(\rho - R_c)$, and Eq. (S57) leads to the solution of the classical induced potential

$$\phi_m^{\text{classical}}(\rho, \omega) = \begin{cases} -2\frac{\pi}{|m|}\frac{\rho^{|m|}}{R_c^{|m|-1}}\sigma_m(\omega), & \text{for } \rho < R_c, \\ \\ -2\frac{\pi}{|m|}\frac{R_c^{|m|+1}}{\rho^{|m|}}\sigma_m(\omega), & \text{for } \rho > R_c. \end{cases} \tag{S58}$$

However, when quantum surface effects are considered within the SRF, $\delta n_m(\rho, \omega)$ is not strictly located at the jellium edge of the nanowire but it extends smoothly near the metal surface. In what follows, we assume that $\delta n_m(\rho, \omega)$ is nonzero only in the small region $\pm\Delta$ inside and outside the classical image plane, which coincides with the jellium edge located at $\rho = R_c$. Thus, Eq. (S57) can be written as

$$\phi_m(\rho, \omega) = \begin{cases} -2\frac{\pi}{|m|}\int_{R_c-\Delta}^{R_c+\Delta}\rho'\, d\rho' \left(\frac{\rho}{\rho'}\right)^{|m|}\delta n_m(\rho', \omega), & \text{for } \rho < R_c - \Delta, \\ \\ -2\frac{\pi}{|m|}\int_{R_c-\Delta}^{R_c+\Delta}\rho'\, d\rho' \left(\frac{\rho'}{\rho}\right)^{|m|}\delta n_m(\rho', \omega), & \text{for } \rho > R_c + \Delta. \end{cases} \tag{S59}$$

Here, we made it explicit that, because of the localization of the induced electron density $\delta n_m(\rho', \omega)$ near the jellium edge, ρ' varies within the interval $\pm\Delta$ around R_c. We can thus develop $\left(\frac{\rho}{\rho'}\right)^{|m|}$ and $\left(\frac{\rho'}{\rho}\right)^{|m|}$ into a Taylor series around R_c with respect to ρ', which results in

$$\left(\frac{\rho}{\rho'}\right)^{|m|} = \frac{\rho^{|m|}}{R_c^{|m|}} - |m|\frac{\rho^{|m|}}{R_c^{|m|+1}}(\rho' - R_c) + \mathcal{O}\left(\frac{(\rho' - R_c)^2}{R_c^2}\right) \approx \frac{\rho^{|m|}}{R_c^{|m|}}\left[1 - |m|\frac{(\rho' - R_c)}{R_c}\right], \tag{S60}$$

and

$$\left(\frac{\rho'}{\rho}\right)^{|m|} = \frac{R_c^{|m|}}{\rho^{|m|}} + |m|\frac{R_c^{|m|-1}}{\rho^{|m|}}(\rho' - R_c) + \mathcal{O}\left(\frac{(\rho' - R_c)^2}{R_c^2}\right) \approx \frac{R_c^{|m|}}{\rho^{|m|}}\left[1 + |m|\frac{(\rho' - R_c)}{R_c}\right]. \tag{S61}$$

Using the Taylor expansion given by Eq. (S60) and Eq. (S61), the radial part of the potential $\phi_m(\rho, \omega)$ within the SRF (given by Eq. (S59)) outside the surface region $R_c \pm \Delta$ can be expressed as

$$\phi_m(\rho, \omega) = \begin{cases} -2\frac{\pi}{|m|}\left(\frac{\rho}{R_c}\right)^{|m|}\int_{R_c-\Delta}^{R_c+\Delta}\rho'\, d\rho'\delta n_m(\rho', \omega)\left[1 - |m|\frac{\rho' - R_c}{R_c}\right], & \text{for } \rho < R_c - \Delta, \\ \\ -2\frac{\pi}{|m|}\left(\frac{R_c}{\rho}\right)^{|m|}\int_{R_c-\Delta}^{R_c+\Delta}\rho'\, d\rho'\delta n_m(\rho', \omega)\left[1 + |m|\frac{\rho' - R_c}{R_c}\right], & \text{for } \rho > R_c + \Delta. \end{cases} \tag{S62}$$

By comparing the potential $\phi_m(\rho, \omega)$ within the SRF given by Eq. (S62) and the classical result $\phi_m^{\text{classical}}(\rho, \omega)$ given by Eq. (S58), one can define the surface electron density,

$$N^{\text{cyl}}(\omega, m) \equiv \int_{R_c-\Delta}^{R_c+\Delta}\rho d\rho\ \delta n_m(\rho, \omega), \tag{S63}$$

and the Feibelman parameter for a cylindrical nanowire,

$$d_{\perp}^{\text{cyl}}(\omega, m) = \frac{\int_{R_c-\Delta}^{R_c+\Delta} \rho \, d\rho \, (\rho - R_c) \, \delta n_m(\rho, \omega)}{\int_{R_c-\Delta}^{R_c+\Delta} \rho \, d\rho \, \delta n_m(\rho, \omega)}, \tag{S64}$$

so that the induced potential $\phi_m(\rho, \omega)$ within the SRF outside the surface region $R_c \pm \Delta$ is given by

$$\phi_m(\rho, \omega) = \begin{cases} \phi_m^{<}(\rho, \omega), & \text{for } \rho < R_c - \Delta, \\ \phi_m^{>}(\rho, \omega), & \text{for } \rho > R_c + \Delta, \end{cases} \tag{S65}$$

where

$$\phi_m^{<}(\rho, \omega) = -2\frac{\pi}{|m|} \left(\frac{\rho}{R_c}\right)^{|m|} N^{\text{cyl}}(\omega, m) \left[1 - \frac{|m|}{R_c} d_{\perp}^{\text{cyl}}(\omega, m)\right], \tag{S66}$$

and

$$\phi_m^{>}(\rho, \omega) = -2\frac{\pi}{|m|} \left(\frac{R_c}{\rho}\right)^{|m|} N^{\text{cyl}}(\omega, m) \left[1 + \frac{|m|}{R_c} d_{\perp}^{\text{cyl}}(\omega, m)\right]. \tag{S67}$$

Note that, for $R_c \to \infty$ or $\Delta \to 0$, Eqs. (S65)–(S67) reduce to the classical local solution given by Eq. (S58) with $\sigma_m(\omega) = \frac{N^{\text{cyl}}(\omega,m)}{R_c}$. In the following subsection we use Eq. (S65), Eq. (S66) and Eq. (S67) to obtain simple solutions of the plasmon resonances of a cylindrical nanowire using the Feibelman parameter $d_{\perp}^{\text{cyl}}(\omega, m)$.

S3.2 Plasmon resonances sustained by a cylindrical nanowire within the SRF

To establish a connection between the SRF solution of the induced potential given by Eqs. (S65)–(S67) and the classical electromagnetic theory, we assume that the finite size 2Δ of the surface region can be neglected and that the classical boundary conditions are retrieved in the form [S19, S20]

$$\hat{\mathbf{n}} \cdot \mathbf{D}^{<}(R_c, \varphi, \omega) = \hat{\mathbf{n}} \cdot \mathbf{D}^{>}(R_c, \varphi, \omega), \tag{S68}$$

where $\hat{\mathbf{n}}$ is the normal unit vector pointing outwards from the metal boundary.

Since $\mathbf{D}(\rho, \varphi, \omega) = \varepsilon_{\mathbf{r}}(\rho, \varphi, \omega)\mathbf{E}(\rho, \varphi, \omega)$ (with $\varepsilon_{\mathbf{r}}$ the spatially-dependent dielectric function), $\hat{\mathbf{n}} = \hat{\rho}$, and $\mathbf{E}(\rho, \varphi, \omega) = \mathbf{E}^{\text{ext}}(\rho, \varphi, \omega) - \nabla\phi(\rho, \varphi, \omega)$ (with $\mathbf{E}^{\text{ext}}(\rho, \varphi, \omega)$ the external electric field) the boundary condition given by Eq. (S68) yields

$$\varepsilon(\omega) \left(\hat{\rho} \cdot \mathbf{E}_m^{\text{ext}}(\rho, \omega) - \frac{\partial \phi_m^{<}(\rho, \omega)}{\partial \rho}\right)\Bigg|_{\rho=R_c} = \left(\hat{\rho} \cdot \mathbf{E}_m^{\text{ext}}(\rho, \omega) - \frac{\partial \phi_m^{>}(\rho, \omega)}{\partial \rho}\right)\Bigg|_{\rho=R_c}, \tag{S69}$$

where $\varepsilon(\omega)$ is the dielectric function of the metal, we assume that there is vacuum outside the metal ($\varepsilon_{\mathbf{r}} = 1$), and $\mathbf{E}_m^{\text{ext}}(\rho, \omega)$ is the m - angular harmonic of the radial component of the external field such that

$$\mathbf{E}^{\text{ext}}(\rho, \varphi, \omega) = \sum_m \mathbf{E}_m^{\text{ext}}(\rho, \omega) e^{im\varphi}. \tag{S70}$$

Using the definitions of the potentials $\phi_m^{<}(\rho, \omega)$ and $\phi_m^{>}(\rho, \omega)$ given by Eq. (S66) and Eq. (S67), we finally obtain the surface electron density of the nanowire as

$$N^{\text{cyl}}(\omega, m) = -\frac{\varepsilon(\omega) - 1}{\varepsilon(\omega) + 1 - \frac{|m|}{R_c} d_{\perp}^{\text{cyl}}(\omega, m) \left[\varepsilon(\omega) - 1\right]} \frac{R_c}{2\pi} E_{m,R_c}^{\text{ext}}(\omega), \tag{S71}$$

where

$$E^{\rm ext}_{m,R_c}(\omega) = \hat{\rho}\cdot\mathbf{E}^{\rm ext}_m(\rho,\omega)\Big|_{\rho=R_c}. \tag{S72}$$

In what follows, we assume that the metal dielectric function $\varepsilon(\omega)$ is well described with a Drude model,

$$\varepsilon(\omega) = 1 - \frac{\omega_p^2}{\omega(\omega+i\gamma_p)}, \tag{S73}$$

where ω_p is the bulk plasma frequency and γ_p the intrinsic damping parameter. Then, from Eq. (S71) we obtain

$$N^{\rm cyl}(\omega,m) = \frac{\omega_p^2}{2\omega(\omega+i\gamma_p) - \omega_p^2\left[1-\frac{|m|}{R_c}d_\perp^{\rm cyl}(\omega,m)\right]}\,\frac{R_c}{2\pi}\,E^{\rm ext}_{m,R_c}(\omega). \tag{S74}$$

The multipolar plasmon resonance frequencies ω_m are given by the poles of the denominator in Eq. (S74),

$$2\omega_m(\omega_m+i\gamma_p) - \omega_p^2\left[1-\frac{|m|}{R_c}d_\perp^{\rm cyl}(\omega,m)\right] = 0. \tag{S75}$$

Thus, for $d_\perp^{\rm cyl}(\omega,m) \ll R_c$, we can use the Taylor expansion and express the frequency of the plasmon resonance as

$$\omega_m = \omega_{\rm SP}\quad\left[1-\frac{1}{2}\frac{|m|}{R_c}{\rm Re}\{d_\perp^{\rm cyl}(\omega=\omega_m,m)\}\right], \tag{S76}$$

and the width as

$$\Gamma_m = \omega_{\rm SP}\quad\frac{|m|}{R_c}{\rm Im}\left\{d_\perp^{\rm cyl}(\omega=\omega_m,m)\right\} + \gamma_p. \tag{S77}$$

where $\omega_{\rm SP} = \frac{\omega_p}{\sqrt{2}}$ is the surface plasmon frequency for $k_\parallel = 0$.

We note that setting $k_\parallel = |m|/R_c$ the above expressions are equivalent to that for the propagating surface plasmon in the planar geometry,

$$\omega_s(k_\parallel) = \omega_{\rm SP}\Big(1-\frac{k_\parallel}{2}{\rm Re}\{d_\perp(\omega_s,k_\parallel)\}\Big), \tag{S78}$$

$$\Gamma_s(k_\parallel) = \omega_{\rm SP}k_\parallel{\rm Im}\{d_\perp(\omega_s,k_\parallel)\} + \gamma_p, \tag{S79}$$

with $d_\perp(\omega_s,k_\parallel)$ being the Feibelman parameter calculated for the planar metal surface. These expressions can also be directly derived from the SRF for the planar surface [S19] (under the assumption $d_\parallel = 0$). Thus, Eq. (S78) and Eq. (S79) with $k_\parallel = k_\parallel^m = |m|/R_c$ can be used instead of Eq. (S76) and Eq. (S77) to obtain the properties of the multipolar plasmons resonances in the cylindrical nanowire.

S4 Multipolar polarizabilities of a cylindrical nanowire

S4.1 General expressions of the multipolar polarizability and multipole moment

In this section, we obtain the expressions of the multipolar polarizabilities and multipole moments of a cylindrical nanowire based on the expression of the induced potential $\phi^{>}(\rho,\varphi,\omega)$ far from the metal surface ($\rho >> R_c$). Using Eq. (S52), Eq. (S57), and Eq. (S65) leads to

$$\phi^{>}(\rho,\varphi,\omega) = \sum_m \phi^{>}_m(\rho,\omega) e^{im\varphi}, \tag{S80}$$

with

$$\phi^{>}_m(\rho,\omega) = -\frac{2\pi}{|m|}\,\frac{1}{\rho^{|m|}} \int_0^{\rho} \rho'\; d\rho'\; \rho'^{|m|}\; \delta n_m(\rho',\omega). \tag{S81}$$

The electron density momentum, $\delta n_m(\rho,\omega)$, is given by (see Eq. (S53))

$$\delta n_m(\rho,\omega) = \frac{1}{2\pi}\int_0^{2\pi} d\varphi e^{-im\varphi} \delta n(\rho,\varphi,\omega), \tag{S82}$$

where $\delta n(\rho,\varphi,\omega)$ is the electron density induced by the external perturbation acting on the system (note that the induced charge density is given by $-\delta n(\rho,\varphi,\omega)$). In practice, the integration in Eq. (S81) runs in the limits $R_c - \Delta \le \rho' \le R_c + \Delta$, since the induced electron density $\delta n(\rho,\varphi,\omega)$ is zero outside this region.

Introducing the multipole moment $Q_m(\omega)$ per unit length of the nanowire as

$$Q_m(\omega) = -\frac{2\pi}{|m|}\int_0^{\infty} \rho\; d\rho\; \rho^{|m|}\; \delta n_m(\rho,\omega), \tag{S83}$$

leads to

$$\phi^{>}_m(\rho,\omega) = \frac{Q_m(\omega)}{\rho^{|m|}}. \tag{S84}$$

The induced potential can then be cast in the form

$$\phi^{>}(\rho,\varphi,\omega) = \sum_m \frac{Q_m(\omega)}{\rho^{|m|}} e^{im\varphi}. \tag{S85}$$

The expression given by Eq. (S85) is valid irrespective of the theoretical (classical, SRF or TDDFT) framework.

Let us introduce next the multipolar polarizability $\alpha_m(\omega)$ per unit length of the nanowire along the z-axis. The multipolar polarizability $\alpha_m(\omega)$ is defined as

$$Q_m(\omega) = \alpha_m(\omega)\; E^{\text{ext}}_{m,\rho}(\omega), \tag{S86}$$

where $E^{\text{ext}}_{m,\rho}(\omega)$ is the m - angular harmonic of the radial component of the external field evaluated at the nanowire surface (given by Eq. (S72)). Note that the free-electron metal cylindrical nanowire is rotationally invariant, so that the response of the system is degenerate for $\pm m$, i.e., $\alpha_m(\omega) = \alpha_{-m}(\omega) = \alpha_{|m|}(\omega)$.

S4.2 Multipolar polarizability within the SRF and the classical theory

We start with the SRF to obtain the multipolar polarizability $\alpha_m^{\mathrm{SRF}}(\omega)$ per unit length of the nanowire along the z-axis. Consider the induced potential at a radial distance $\rho >> R_c$ significantly larger than the nanowire radius so that the induced electron density of the nanowire is zero. Using Eq. (S67), Eq. (S71), Eq. (S84) and Eq. (S86) it follows that

$$\alpha_{|m|}^{\mathrm{SRF}}(\omega) = \frac{R_c^{|m|+1}}{|m|} \, \frac{\varepsilon(\omega) - 1}{\varepsilon(\omega) + 1 - [\varepsilon(\omega) - 1] \, \frac{|m|}{R_c} \, d_\perp^{\mathrm{cyl}}(\omega, |m|)} \left[1 + \frac{|m|}{R_c} \, d_\perp^{\mathrm{cyl}}(\omega, |m|)\right]. \tag{S87}$$

It can be observed in Eq. (S87) that the quantity $\alpha_{|m|}^{\mathrm{SRF}}/R_c^{|m|}$ for a fixed value $|m|/R_c$ is independent of the radius of the nanowire as long as $d_\perp^{\mathrm{cyl}}(\omega, |m|)$ is a function of the effective wavenumber $k_\parallel^m = |m|/R_c$ rather than of $|m|$, which is indeed the case as demonstrated in Section S5. As a consequence, the quantity $\alpha_{|m|}^{\mathrm{SRF}}/R_c^{|m|}$ obtained e.g. for a nanowire of radius $R_c = 75$ a$_0$ and $m = 1$ is identical within the SRF to that obtained for $R_c = 150$ a$_0$ and $m = 2$, as we confirm below in this section using TDDFT.

The expression for the multipolar polarizability within the classical theory, $\alpha_m^{\mathrm{class}}(\omega)$, can be obtained from Eq. (S87) by setting $d_\perp^{\mathrm{cyl}} = 0$, which leads to

$$\alpha_{|m|}^{\mathrm{class}}(\omega) = \frac{R_c^{|m|+1}}{|m|} \, \frac{\varepsilon(\omega) - 1}{\varepsilon(\omega) + 1}. \tag{S88}$$

According to Eq. (S88), the quantity $\alpha_{|m|}^{\mathrm{class}}/R_c$ for a fixed value $|m|/R_c^{|m|}$ is independent of the radius of the nanowire similarly to what we obtained within the SRF.

S4.3 Multipolar polarizability from TDDFT calculations

Within TDDFT, we calculate the density response of the system to an impulsive external potential $V^{\mathrm{ext}}(\rho, \varphi, t)$ with the time-dependence given by the Dirac delta function, $\delta(t)$,

$$V^{\mathrm{ext}}(\rho, \varphi, t) = -\xi \; \delta(t) \sum_{m=1}^{m_{\max}} \left(\frac{\rho}{R_c}\right)^m \cos(m\varphi), \tag{S89}$$

where the lower summation limit $m = 1$ indicates that this excitation only involves harmonic contributions with *positive* magnetic quantum numbers, $m \geq 1$. The amplitude of the potential, ξ, is sufficiently weak to ensure a linear response. The upper summation limit, $m_{\max}$, sets the highest multipole moment excited in the system. It is worth noting here that the external potential $V_{\mathrm{ext}}(x, y, t)$ entering in the KS Hamiltonian in TDDFT calculations (see Eq. (S47)) is given by $-V^{\mathrm{ext}}(\rho, \varphi, t)$ (see Eq. (S89)) expressed in Cartesian coordinates. The minus sign stands for the electron charge.

The relationship between the external time-dependent potential $V^{\mathrm{ext}}(\rho, \varphi, t)$ given by Eq. (S89) and the external time-dependent electric field $\mathbf{E}^{\mathrm{ext}}(\rho, \varphi, t)$ is (see Eq. (S70)):

$$\mathbf{E}^{\mathrm{ext}}(\rho, \varphi, t) = \sum_m \mathbf{E}_m^{\mathrm{ext}}(\rho, t) e^{im\varphi} = -\nabla V^{\mathrm{ext}}(\rho, \varphi, t) = -\nabla \sum_m V_m^{\mathrm{ext}}(\rho, t) e^{im\varphi}, \tag{S90}$$

where the external potential has also been expressed as a summation of different m-angular harmonics, $V^{\rm ext}(\rho,\varphi,t) = \sum_m V_m^{\rm ext}(\rho,t)e^{im\varphi}$. Since $\cos(m\varphi) = \frac{1}{2}\left[e^{im\varphi} + e^{-im\varphi}\right]$ we obtain from Eq. (S89)

$$V_m^{\rm ext}(\rho,t) = -\frac{\xi}{2}\,\delta(t)\,\left(\frac{\rho}{R_c}\right)^{|m|}. \tag{S91}$$

The frequency-resolved external potential is then given by

$$V^{\rm ext}(\rho,\varphi,\omega) = \sum_m V_m^{\rm ext}(\rho,\omega)e^{im\varphi}, \tag{S92}$$

where $V_m^{\rm ext}(\rho,\omega)$ is obtained from the time-to-frequency Fourier transform of $V_m^{\rm ext}(\rho,t)$

$$V_m^{\rm ext}(\rho,\omega) = \int_{-\infty}^{\infty} e^{i\omega t}V_m^{\rm ext}(\rho,t)dt = -\frac{\xi}{2}\,\left(\frac{\rho}{R_c}\right)^{|m|}. \tag{S93}$$

Therefore, from Eq. (S72), Eq. (S90) and Eq. (S93) we obtain

$$E^{\rm ext}_{m,R_c}(\omega) = |m|\frac{\xi}{2}\frac{1}{R_c}. \tag{S94}$$

Using the time-dependent induced electron density $\delta n(x,y,t)$ obtained from real-time TDDFT calculations performed in Cartesian coordinates and introduced above, we calculate the following time-dependent quantity ($1 \le m \le m_{\rm max}$)

$$\mathcal{Q}_m(t) = -\frac{1}{m}\iint dx\;dy\;\left(\frac{\rho}{R_c}\right)^{m}\;\cos(m\varphi)\;\delta n(x,y,t). \tag{S95}$$

Here, the "$-$" sign accounts for the electron charge, and the induced electron density $\delta n(x,y,t)$ is given by

$$\delta n(x,y,t) = n(x,y,t) - n(x,y,t=0). \tag{S96}$$

Because of the external potential the induced density develops in $\cos(m\varphi)$ series, we can write

$$\begin{aligned}\mathcal{Q}_m(t) &= -2\pi\frac{1}{m}\iint dx\;dy\;\left(\frac{\rho}{R_c}\right)^{m}\;\frac{e^{-im\varphi}}{2\pi}\;\delta n(x,y,t)\\ &= -2\pi\frac{1}{m}\int_0^{\infty}\rho d\rho\;\left(\frac{\rho}{R_c}\right)^{m}\;\int_0^{2\pi}d\varphi\frac{e^{-im\varphi}}{2\pi}\;\delta n(\rho,\varphi,t)\\ &= -2\pi\frac{1}{m}\int_0^{\infty}\rho d\rho\;\left(\frac{\rho}{R_c}\right)^{m}\;\delta n_m(\rho,t).\end{aligned} \tag{S97}$$

The frequency-resolved spectrum of $\mathcal{Q}_m(t)$ is finally obtained from the time-to-frequency Fourier transform,

$$\begin{aligned}\mathcal{Q}_m(\omega) &= \int dt\;\mathcal{Q}_m(t)e^{(i\omega-\eta/2)t},\\ &= -\frac{2\pi}{m}\int_0^{\infty}\rho d\rho\;\left(\frac{\rho}{R_c}\right)^{m}\;\delta n_m(\rho,\omega).\end{aligned} \tag{S98}$$

The attenuation factor $\eta = 0.15$ eV is introduced to mimic dissipation processes beyond the reach of the ALDA-TDDFT scheme adopted here [S21, S22, S23, S24].

By comparing Eq. (S83) and Eq. (S98) we obtain that the multipole moment $Q_m(\omega)$ per unit length is

$$Q_m(\omega) = \mathcal{Q}_m(\omega) R_c^m. \tag{S99}$$

Finally, using Eq. (S86), Eq. (S94), and Eq. (S99), the polarizability $\alpha_m^{\text{TDDFT}}(\omega)$ within TDDFT can be obtained from

$$\alpha_m^{\text{TDDFT}}(\omega) = \frac{2}{\xi} \frac{R_c}{m} R_c^m \mathcal{Q}_m(\omega), \tag{S100}$$

where $\mathcal{Q}_m(\omega)$ is obtained from the TDDFT calculations of the induced electron density by applying Eq. (S98). The ratio $\alpha_m^{\text{TDDFT}}/R_c^{|m|}$ for a fixed value $|m|/R_c$ is expected to be independent of the radius of the nanowire in analogy with our findings within the SRF. Thus, it can be expected that $\mathcal{Q}_m(\omega)$ obtained from Eq. (S98) is almost independent of the nanowire radius R_c once the quantity $|m|/R_c$ is fixed (see Eq. (S100)). In this case, only finite-size effects (strengthened with reducing nanowire size) associated to the discrete electronic levels of the metal can lead to slightly different results of $\mathcal{Q}_m(\omega)$ for nanowires of different radii but fixed value $|m|/R_c$. The normalization $\alpha_m^{\text{TDDFT}}/R_c^{|m|}$ thus simplifies the comparison between the results of the calculations performed for nanowires of different radii Rc, and allows to benchmark different theories using TDDFT.

We compare in Fig. S1 the values of $|\alpha_m^{\text{TDDFT}}(\omega)/R_c^m)|^2$ obtained with TDDFT for nanowires with radii $R_c = 75$ a_0 (dahsed lines), 100 a_0 (dotted lines), and 150 a_0 (solid lines). Results are shown for selected values of m such that $k_{\parallel}^m = m/R_c = 0.013,\ 0.02,\ 0.04,\ 0.067,$ and $0.1\ a_0^{-1}$. When the $1/R_c^m$ normalization is performed, results obtained for nanowires of different size show near perfect match with each other once the ratio m/R_c is fixed. This provides further confirmation on the role of an effective wavenumber $k_{\parallel}^m = m/R_c$ in determining the optical response of the nanowire in perfect agreement with Eq. (S87).

S5 Assessing the robustness of the dispersive Feibelman parameter

In this section, we compare the dispersive Feibelman parameter $d_\perp$ obtained from frequency-domain TDLDA calculations for the planar metal surface represented within the slab geometry (Section S1, Eq. (S38)) and the results obtained from real-time TDDFT for cylindrical nanowires of different radius R_c (Section S2, Eq. (S64)).

S5.1 Real-time TDDFT calculation of $d_\perp^{\text{cyl}}(\omega, m)$

As discussed in the main text and in Section S2 of this Supplementary Material, the m multipolar electron density at the surface of the cylindrical nanowire is well represented by the dynamics of the density distribution characterized by the effective wavenumber $k_{\parallel}^m = |m|/R_c$ at the planar metal surface. Indeed, for large R_c one expects equivalence between the two geometries. Thus, instead of $d_\perp^{\text{cyl}}(\omega, m)$ given by Eq. (S64), one can use the Feibelman parameter calculated for the planar metal surface for $k_{\parallel} = k_{\parallel}^m = |m|/R_c$, i.e., $d_\perp(\omega, m/R_c)$, defined by Eq. (S38) (Eq. (4) in the main text). In Fig. 3b of the main text, we demonstrate that this approach allows

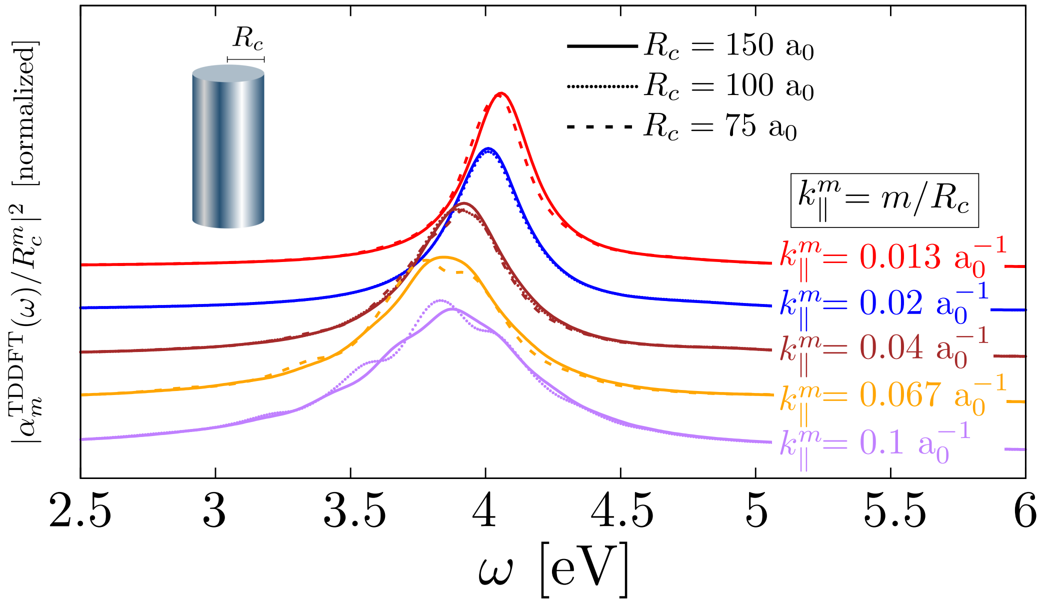

Figure S1: Multipolar polarizabilities $|\alpha_m^{\rm TDDFT}(\omega)/R_c^m|^2$ obtained with TDDFT for cylindrical nanowires of different radii R_c and selected values of the magnetic number m. Results are shown as function of frequency. Solid lines: $R_c = 150$ a$_0$. Dotted lines: $R_c = 100$ a$_0$. Dashed lines: $R_c = 75$ a$_0$. The selected values of m are such that the effective wavenumber $k_\parallel^m = m/R_c = 0.013$ a$_0^{-1}$ (red), $k_\parallel^m = 0.02$ a$_0^{-1}$ (blue), $k_\parallel^m = 0.04$ a$_0^{-1}$ (brown), $k_\parallel^m = 0.067$ a$_0^{-1}$ (orange), and $k_\parallel^m = 0.1$ a$_0^{-1}$ (purple) for the three values of R_c. Results are vertically offset for clarity.

one to describe the energies of the (localized) multipolar plasmons of the nanowire over the broad range of plasmonic order m. In what follows, we further test the above finding by directly comparing the Feibelman parameter calculated for the planar metal surface and that obtained from Eq. (S64) for the cylindrical nanowires of different R_c.

The real-time TDDFT methodology introduced in Section S2 can be used to compute the Feibelman parameter $d_\perp^{\rm cyl}(\omega, m)$ of the nanowire given by Eq. (S64). In practice, to calculate $d_\perp^{\rm cyl}(\omega, m)$ within the TDDFT approach in Cartesian coordinates (see Section S2), we first compute the following two time-dependent quantities

$$\mathcal{P}_\perp(t, m) = \iint \cos(m\varphi)\ \delta n(x, y, t)\ \rho\ (\rho - R_c)\ dxdy,$$
$$\mathcal{Q}_\perp(t, m) = \iint \cos(m\varphi)\ \delta n(x, y, t)\ \rho\ dxdy, \tag{S101}$$

where $\delta n(x, y, t)$ is defined by Eq. (S96).

The time-to-frequency Fourier transform (see Eq. (S98)) is then used to obtain $\mathcal{P}_\perp(\omega, m)$ and $\mathcal{Q}_\perp(\omega, m)$. Then,

$$d_\perp^{\rm cyl}(\omega, m) = \frac{\mathcal{P}_\perp(\omega, m)}{\mathcal{Q}_\perp(\omega, m)}. \tag{S102}$$

Note that, in difference with Eq. (S64), our numerical procedure operates with integrand quantities multiplied by the radial coordinate ρ. Since the integrals are accumulated within the

surface region where $\rho \approx R_c$, and since $d_\perp^{\rm cyl} \ll R_c$, the resulting value of $d_\perp^{\rm cyl}$ is not affected as we explicitly checked. We found, however, that this procedure improves the numerical convergence by focusing explicitly at the surface region.

S5.2 Comparison between the dispersive Feibelman parameter $d_\perp^{\rm cyl}(\omega, m)$ calculated with TDDFT for different nanowire sizes and $d_\perp(\omega, k_\parallel)$ calculated with TDLDA for the planar metal surface.

In this subsection, we analyze the dispersive Feibelman parameter obtained for nanowire and planar geometries. Fig. S2 shows the real (panel a) and imaginary (b) parts of $d_\perp^{\rm cyl}(\omega, m)$ calculated with TDDFT for nanowires of radius $R_c = 100$ a$_0$ and $R_c = 150$ a$_0$. The values of m are chosen independently for each nanowire size R_c in such a way that the ratio $|m|/R_c$ is fixed. Results in Fig. S2 correspond to $|m|/R_c = 0.02,\ 0.1$ and 0.2 a$_0^{-1}$. In the main text, we argue that the effective wavenumber $k_\parallel^m = |m|/R_c$ determines the optical response of the system. This finding is further supported by the TDDFT calculations in Fig. S2, where $d_\perp^{\rm cyl}(\omega, m)$ obtained with different R_c are nearly the same as long as the ratio $k_\parallel^m = |m|/R_c$ is fixed. The oscillating features in the frequency dependence of $d_\perp^{\rm cyl}$ arise because of the finite-size effects, which differ for different radius R_c. The results in Fig. S2 thus further confirm that the effective wavenumber $k_\parallel^m = |m|/R_c$ is the key parameter determining the surface screening and multipolar plasmon modes in the cylindrical nanowire, which justifies using $d_\perp(\omega, k_\parallel)$ obtained from the TDLDA study for the planar metal surface as employed in the main text.

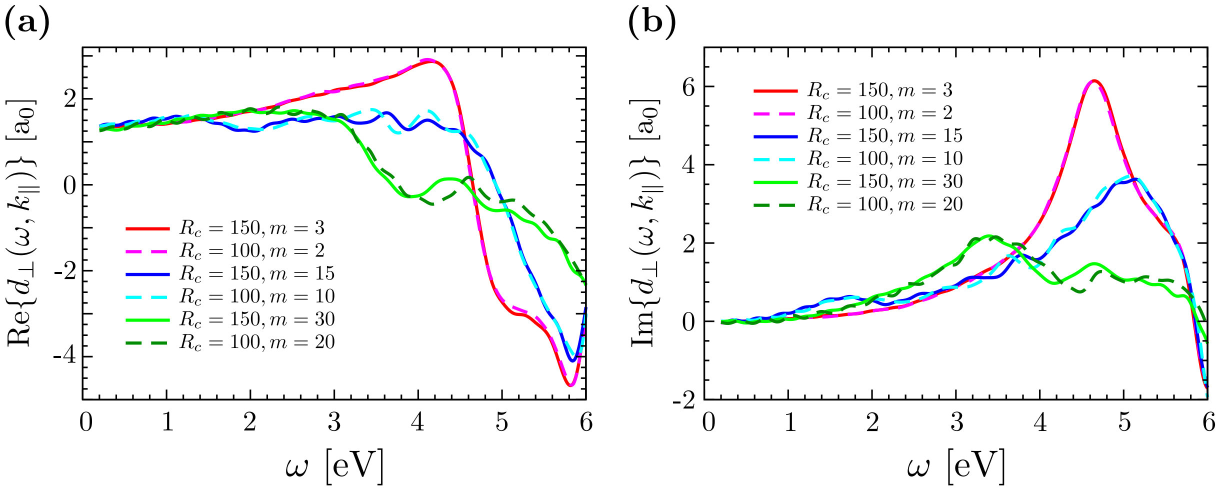

Figure S2: Comparison of Feibelman parameters obtained for cylindrical nanowires with different radii. **(a)** Real part of the dispersive Feibelman parameter $d_\perp^{\rm cyl}(\omega, m)$ for different values of m and R_c, as obtained from TDDFT calculations performed for cylindrical nanowires. Results are shown as a function of the frequency, ω. The values of m and R_c are indicated in the inset. For cylinders of different radii m is chosen in such a way that the ratio $k_\parallel^m = |m|/R_c$ is fixed to $|m|/R_c = 0.02,\ 0.1$ and 0.2 a$_0^{-1}$. **(b)** Same as in (a) but for the imaginary part.

To further stress this finding, we directly compare in Fig. S3 the Feibelman parameter $d_\perp(\omega, k_\parallel)$ calculated within TDLDA for the metal slab and $d_\perp^{\rm cyl}(\omega, m)$ obtained within TDDFT

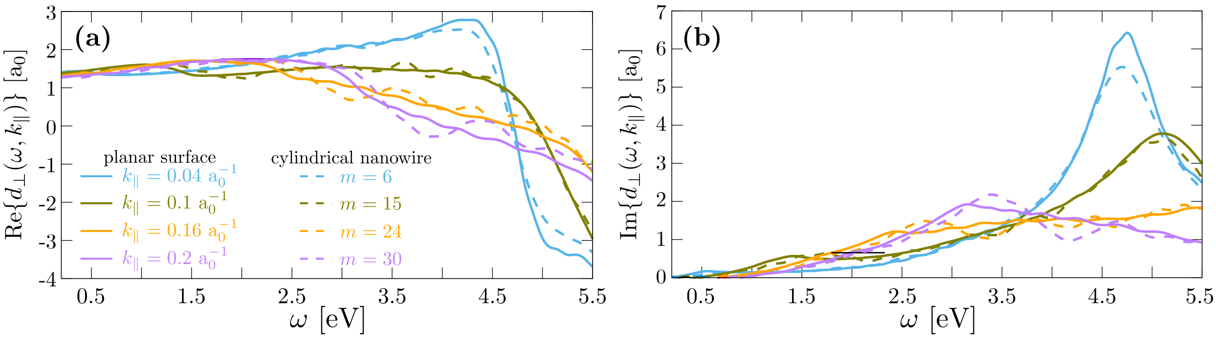

Figure S3: Comparison of Feibelman parameters obtained for cylindrical and planar surface geometries. **(a)** Solid lines: Real part of $d_\perp(\omega, k_\|)$ calculated with frequency-domain TDLDA for a metal slab of a thickness $L = 870$ a_0 representing the planar metal surface. Results are shown for $k_\| = 0.04$ a_0^{-1}, 0.1 a_0^{-1}, 0.16 a_0^{-1}, and 0.2 a_0^{-1}. Dashed lines: Real part of the dispersive Feibelman parameter $d_\perp^{\rm cyl}(\omega, m)$ calculated with real-time TDDFT for a cylindrical nanowire of radius $R_c = 150$ a_0 and $m = 6, 15, 24, 20$. Results are shown as a function of the frequency, ω. **(b)** Same as in (a) but for the imaginary part.

for the cylindrical nanowire. As long as the wavevector $k_\|$ in the slab is fixed to the ratio $k_\|^m = |m|/R_c$ in the nanowire, $k_\| = k_\|^m$, the results obtained with different geometries are very close to each other. Note also that, because of the larger size of the metal slab as compared to the cylindrical nanowire, the features associated with finite-size effects are significantly smaller for the slab. The frequency dependence of the Feibelman parameters appears less structured and smoother as compared to the cylindrical geometry.

S6 Resonant frequency and width of the plasmon resonances

In this section, we provide a more detailed analysis of the resonant frequencies and width of the plasmon resonances studied in this work. The resonant frequencies $\omega_s(k_\parallel)$ of the surface plasmon sustained by the planar metal slab are obtained from Eq. (6) in the main text (or Eq. (S78) in this Supplementary Material). This equation is obtained from the Taylor expansion truncated at the first-derivative order of a more general equation given by:

$$\omega_s(k_\parallel) = \omega_{\mathrm{SP}}\sqrt{1 - k_\parallel \mathrm{Re}\{d_\perp(\omega_s, k_\parallel)\}}, \tag{S103}$$

and thus assumes that $|k_\parallel \mathrm{Re}\{d_\perp(\omega_s, k_\parallel)\}| << 1$. Within the dispersive SRF proposed in this work, we obtain a maximum value of $|k_\parallel \mathrm{Re}\{d_\perp(\omega_s, k_\parallel)\}| = 0.15$, while within the nondispersive SRF we obtain a maximum value of $|k_\parallel \mathrm{Re}\{d_\perp(\omega_s, k_\parallel = 0)\}| = 0.45$. The maximum value within the dispersive SRF is considerably smaller than the non-dispersive one since, in the former case, the value of $\mathrm{Re}\{d_\perp(\omega = \omega_s, k_\parallel)\}$ decreases with increasing $k_\parallel$ as shown in Figure 2a of the manuscript.

We analyze in Figure S4 the validity of the assumption ($|k_\parallel \mathrm{Re}\{d_\perp(\omega_s, k_\parallel)\}| << 1$) adopted in the main text. On the one hand, there is excellent agreement between the results obtained from Eq. (S103) (dark red solid line) and Eq. (S78) (dashed red line) within the dispersive SRF, which indicates that, in this case, assuming $|k_\parallel \mathrm{Re}\{d_\perp(\omega_s, k_\parallel)\}| << 1$ is valid. On the other hand, within the nondispersive SRF model, there are slight differences between the results obtained from Eq. (S103) (dark green solid line) and Eq. (S78) (dashed green line) for $k_\parallel \gtrsim 0.10$ a_0^{-1}. However, these differences are small and the discussion and conclusions of the main text remain valid.

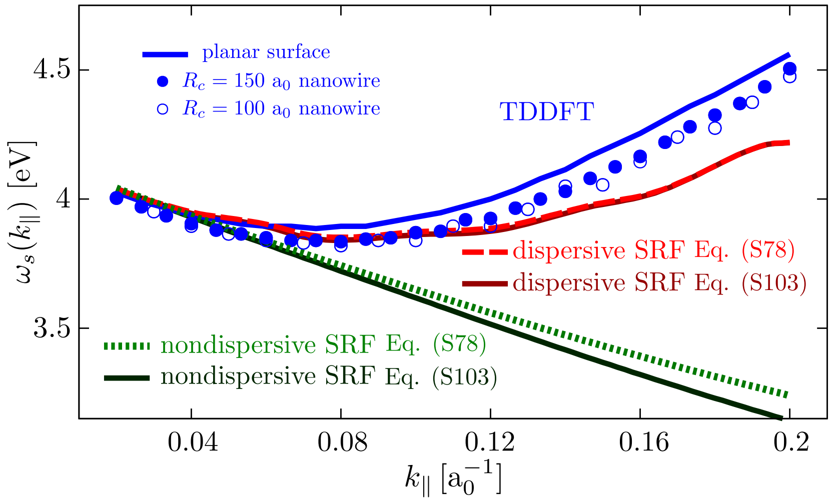

Figure S4: Surface plasmon frequency ω_s, as a function of $k_\parallel$. Blue solid line: linear-response frequency-domain TDLDA results. Red dashed line: dispersive SRF results, as obtained from the perturbative equation given by Eq. (S78). Dark red solid line: dispersive SRF results, as obtained from the nonperturbative equation given by Eq. (S103). Green dotted line: nondispersive SRF results, as obtained from the perturbative equation given by Eq. (S78). Dark green solid line: nondispersive SRF results, as obtained from the nonperturbative equation given by Eq. (S103). We also show the frequency ω_m of the localized multipolar plasmon of order m sustained by a cylindrical metallic nanowire of radius $R_c = 150$ a$_0$ (filled circles) and $R_c = 100$ a$_0$ (hollow circles) as a function of the effective wavenumber $k_\parallel^m = |m|/R_c$.

Finally, we analyze in Fig. S5 the width $\Gamma_s(k_\parallel)$ of plasmon resonances sustained by the planar metal surface as a function of $k_\parallel$, obtained within TDLDA (blue solid line), the dispersive SRF (red dashed line), and the nondispersive SRF (green dotted line). Further, we also show the width of the (localized) plasmon resonances of order m sustained by cylindrical nanowires of radius $R_c = 150$ a$_0$ (filled blue circles) and $R_c = 100$ a$_0$ (hollow blue circles) as a function of $k_\parallel = |m|/R_c$. Within TDLDA (used for the planar geometry), we define $\Gamma_s(k_\parallel)$ as the full width at half maximum (FWHM) of the surface loss function $\mathrm{Im}\{g(\omega, k_\parallel)\}$ displayed in Fig. 3a of the main text. Within the dispersive SRF, we obtain $\Gamma_s(k_\parallel)$ from Eq. (S79) using a damping parameter $\gamma_p = 0.1$ eV and the dispersive Feibelman parameter $d_\perp(\omega, k_\parallel)$ calculated in this work for the planar metal surface. The nondispersive SRF results are also obtained using $\gamma_p = 0.1$ eV in Eq. (S79), but in this case we use the nondispersive Feibelman parameter $d_\perp(\omega)$ calculated by Christensen *et al.* [S19] under the long-wavelength limit ($k_\parallel = 0$). The results for the (localized) plasmon resonances in the cylindrical nanowire are obtained as the FWHM of $|\alpha_m^{\mathrm{TDDFT}}(\omega)/R_c^m|^2$ (displayed e.g. in Fig. S1).

The TDLA results of the planar metal slab and the dispersive SRF results are in good agreement, with both predicting a continuous increase of $\Gamma_s(k_\parallel)$ with increasing $k_\parallel$ due to surface-enabled Landau damping (see Eq. (S79)). On the other hand, the nondispersive SRF underestimates the value of $\Gamma_s(k_\parallel)$ at large $k_\parallel \gtrsim 0.08$ a$_0^{-1}$, since $\mathrm{Im}\{d_\perp(\omega_s)\}$ obtained by Christensen *et al.* [S19] is evaluated at plasmon frequencies $\omega_s(k_\parallel)$ that decrease as $k_\parallel$ increases (see Fig. 3b in the main text), effectively reducing the value of $\Gamma_s(k_\parallel)$ according to Eq. (S79) (see Fig. 2b in the main text). Finally, the width of the (localized) plasmon resonances of order m sustained

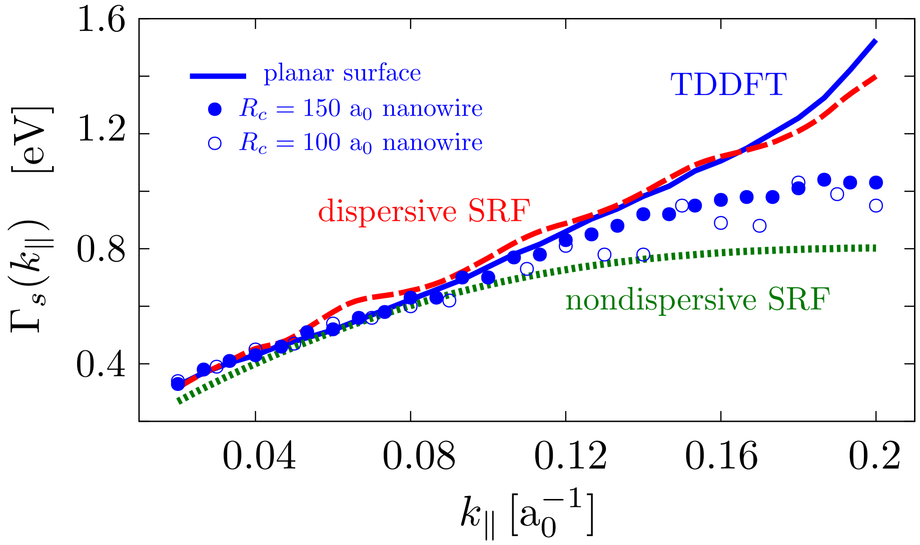

Figure S5: Width of the plasmon resonances, $\Gamma_s(k_\parallel)$, as a function of the wavenumber parallel to the surface, $k_\parallel$. Blue solid line: linear-response frequency-domain TDLDA results corresponding to the planar metal surface, defined as the FWHM of $\text{Im}\{g(\omega, k_\parallel)\}$ displayed in Fig. 3a of the main text. Red dashed line: dispersive SRF results, where the Feibelman parameter $d_\perp(\omega, k_\parallel)$ depends on ω and $k_\parallel$. Green dotted line: nondispersive SRF results, where $d_\perp(\omega)$ is a function of frequency only. Both dispersive and nondispersive SRF results are obtained from Eq. (S79) using an intrinsic damping parameter $\gamma_p = 0.1$ eV. We also show the width of the localized multipolar plasmon resonance of order m sustained by a cylindrical metallic nanowire of radius $R_c = 150$ a$_0$ (filled circles) and $R_c = 100$ a$_0$ (hollow circles) as a function of the effective wavenumber $k_\parallel^m = |m|/R_c$.

by cylindrical nanowires deviates from the results of the planar surface for large $k_\parallel^m \gtrsim 0.14$ a$_0^{-1}$. We attribute this discrepancy between the planar surface and cylindrical nanowire results to finite-size effects that make $|\alpha_m^{\text{TDDFT}}(\omega)/R_c^m|^2$ not to follow a perfect Lorentzian profile for high $k_\parallel$ due to the small radius R_c of the nanowire.

References

[S1] W Ekardt. Dynamical polarizability of small metal particles: self-consistent spherical jellium background model. *Physical review letters*, 52(21):1925, 1984.

[S2] W Ekardt. Work function of small metal particles: Self-consistent spherical jellium-background model. *Physical Review B*, 29(4):1558, 1984.

[S3] Quantum size effects in the electronic properties of small metal particles: Self-consistent spherical jellium background model. *Surface Science*, 152-153:180–188, 1985. ISSN 0039-6028.

[S4] Matthias Brack. The physics of simple metal clusters: self-consistent jellium model and semiclassical approaches. *Reviews of modern physics*, 65(3):677, 1993.

[S5] M Koskinen, PO Lipas, and M Manninen. Electron-gas clusters: the ultimate jellium model. *Zeitschrift für Physik D Atoms, Molecules and Clusters*, 35(4):285–297, 1995.

[S6] Olle Gunnarsson and Bengt I Lundqvist. Exchange and correlation in atoms, molecules, and solids by the spin-density-functional formalism. *Physical Review B*, 13(10):4274, 1976.

[S7] Ansgar Liebsch. *Electronic excitations at metal surfaces*. Springer Science & Business Media, 1997.

[S8] Adolfo G Eguiluz. Self-consistent static-density-response function of a metal surface in density-functional theory. *Physical Review B*, 31(6):3303, 1985.

[S9] Tatiana V Teperik, Peter Nordlander, Javier Aizpurua, and Andrei G Borisov. Robust subnanometric plasmon ruler by rescaling of the nonlocal optical response. *Physical review letters*, 110(26):263901, 2013.

[S10] Tatiana V. Teperik, Peter Nordlander, Javier Aizpurua, and Andrei G. Borisov. Quantum effects and nonlocality in strongly coupled plasmonic nanowire dimers. *Opt. Express*, 21 (22):27306–27325, Nov 2013.

[S11] Garikoitz Aguirregabiria, Dana-Codruta Marinica, Markus Ludwig, Daniele Brida, Alfred Leitenstorfer, Javier Aizpurua, and Andrey G Borisov. Dynamics of electron-emission currents in plasmonic gaps induced by strong fields. *Faraday discussions*, 214:147–157, 2019.

[S12] Markus Ludwig, Garikoitz Aguirregabiria, Felix Ritzkowsky, Tobias Rybka, Dana Codruta Marinica, Javier Aizpurua, Andrei G Borisov, Alfred Leitenstorfer, and Daniele Brida. Sub-femtosecond electron transport in a nanoscale gap. *Nature Physics*, 16(3):341–345, 2020.

[S13] Dana Codruta Marinica, Mario Zapata, Peter Nordlander, Andrey K Kazansky, Pedro M Echenique, Javier Aizpurua, and Andrei G Borisov. Active quantum plasmonics. *Science advances*, 1(11):e1501095, 2015.

[S14] MD Feit, JA Fleck Jr, and A Steiger. Solution of the schrödinger equation by a spectral method. *Journal of Computational Physics*, 47(3):412–433, 1982.

[S15] MD Feit and JA Fleck Jr. Solution of the schrödinger equation by a spectral method ii: Vibrational energy levels of triatomic molecules. *The Journal of Chemical Physics*, 78(1): 301–308, 1983.

[S16] André D Bandrauk and Hai Shen. Improved exponential split operator method for solving the time-dependent schrödinger equation. *Chemical physics letters*, 176(5):428–432, 1991.

[S17] C Clay Marston and Gabriel G Balint-Kurti. The fourier grid hamiltonian method for bound state eigenvalues and eigenfunctions. *The Journal of chemical physics*, 91(6):3571–3576, 1989.

[S18] Ronnie Kosloff. Quantum molecular dynamics on grids. *Dynamics of molecules and chemical reactions*, pages 185–230, 1996.

[S19] Thomas Christensen, Wei Yan, Antti-Pekka Jauho, Marin Soljačić, and N Asger Mortensen. Quantum corrections in nanoplasmonics: shape, scale, and material. *Physical review letters*, 118(15):157402, 2017.

[S20] Yi Yang, Di Zhu, Wei Yan, Akshay Agarwal, Mengjie Zheng, John D Joannopoulos, Philippe Lalanne, Thomas Christensen, Karl K Berggren, and Marin Soljačić. A general theoretical and experimental framework for nanoscale electromagnetism. *Nature*, 576 (7786):248–252, 2019.

[S21] G. Vignale and Walter Kohn. Current-dependent exchange-correlation potential for dynamical linear response theory. *Phys. Rev. Lett.*, 77:2037–2040, Sep 1996.

[S22] Giovanni Vignale, Carsten A Ullrich, and Sergio Conti. Time-dependent density functional theory beyond the adiabatic local density approximation. *Physical review letters*, 79(24): 4878, 1997.

[S23] H. O. Wijewardane and C. A. Ullrich. Time-dependent kohn-sham theory with memory. *Phys. Rev. Lett.*, 95:086401, Aug 2005.

[S24] Carsten A. Ullrich. *Time-dependent density-functional theory: concepts and applications.* Oxford University Press, 2013.
